# Increased emergency cardiovascular events among under-40 population in Israel during vaccine rollout and third COVID-19 wave

**DOI:** 10.1038/s41598-022-10928-z

**Published:** 2022-04-28

**Authors:** Christopher L. F. Sun, Eli Jaffe, Retsef Levi

**Affiliations:** 1 Sloan School of Management, Massachusetts Institute of Technology, 100 Main Street, Cambridge, MA 02142-1347 USA; 2 Healthcare Systems Engineering, Massachusetts General Hospital, Boston, MA USA; 3 Israel National Emergency Medical Services (Magen David Adom), Tel Aviv-Jaffo, Israel; 4 Ben Gurion University of the Negev, Beer Sheva, Israel

**Keywords:** Health policy, Public health, Infectious diseases

## Abstract

Cardiovascular adverse conditions are caused by coronavirus disease 2019 (COVID-19) infections and reported as side-effects of the COVID-19 vaccines. Enriching current vaccine safety surveillance systems with additional data sources may improve the understanding of COVID-19 vaccine safety. Using a unique dataset from Israel National Emergency Medical Services (EMS) from 2019 to 2021, the study aims to evaluate the association between the volume of cardiac arrest and acute coronary syndrome EMS calls in the 16–39-year-old population with potential factors including COVID-19 infection and vaccination rates. An increase of 25% was detected in both call types during January–May 2021, compared with the years 2019–2020. Using Negative Binomial regression models, the weekly emergency call counts were significantly associated with the rates of 1st and 2nd vaccine doses administered to this age group but were not with COVID-19 infection rates. While not establishing causal relationships, the findings raise concerns regarding vaccine-induced undetected severe cardiovascular side-effects and underscore the already established causal relationship between vaccines and myocarditis, a frequent cause of unexpected cardiac arrest in young individuals. Surveillance of potential vaccine side-effects and COVID-19 outcomes should incorporate EMS and other health data to identify public health trends (e.g., increased in EMS calls), and promptly investigate potential underlying causes.

## Introduction

Cardiovascular adverse outcomes such as blood clotting (e.g., coronary artery thrombosis), acute coronary syndrome, cardiac arrest and myocarditis have been identified as consequences of coronavirus disease 2019 (COVID-19) infection^1–5^. Similarly, data from regulatory surveillance and self-reporting systems, including the Vaccine Adverse events Reporting System (VAERS) in the United States (US)^6^, the Yellow Card System in the United Kingdom^7^ and the EudraVigilance system in Europe^8^, associate similar cardiovascular side-effects^9–13^ with a number of COVID-19 vaccines currently in use.

More recently, several studies established probable causal relationship between the messenger RNA (mRNA) vaccines of BNT162b2 and mRNA-1273^11,14–16^ as well as adenovirus (ChAdOx1) vaccines^17^ with myocarditis, primarily in children, young and middle-age adults. The study by the Ministry of Health in Israel, a country with one of the highest vaccination rates in the world, assesses the risk of myocarditis after receiving the 2nd vaccine dose to be between 1 in 3000 to 1 in 6000 in men of age 16–24 and 1 in 120,000 in men under 30^11–13^. A follow up study by the US Center of Disease Control (CDC) based on the VAERS and V-Safe self-reporting systems^18^ further confirms these findings^19^. The CDC has recently posted a warning regarding a vaccine-related risk of myocarditis, but still maintained their recommendation to vaccinate young individuals and children over 12^7^. Similar concerns are reflected in the recent Food and Drug Administration approval to the Pfizer vaccine that requires several follow studies on the short and long terms effects of myocarditis in young individuals^20^.

While the benefits of COVID-19 vaccination are clear, especially for populations at great risk of developing serious and potentially life-threatening illness^15,21^, it is important to better understand the potential risks to minimize potential harm. However, assessing the connection between myocarditis and other potential cardiovascular conditions, and the COVID-19 vaccines is challenging. First, self-reporting systems^22^ of adverse events are known to have self-reporting bias and both under and over-reporting problems^23–25^. Even the study from Israel that is based on more proactive data collection mentions that some of the potentially relevant cases were not fully investigated.

Second, myocarditis is a particularly insidious disease with multiple reported manifestations. There is vast literature that highlights asymptomatic cases of myocarditis, which are often underdiagnosed^26,27^, as well as cases in which myocarditis can possibly be misdiagnosed as acute coronary syndrome (ACS)^28–30^. Moreover, several comprehensive studies demonstrate that myocarditis is a major cause of sudden, unexpected deaths in adults less than 40 years of age, and assess that it is responsible for 12–20% of these deaths^26,31–33^. Thus, it is a plausible concern that increased rates of myocarditis among young people could lead to an increase in other severe cardiovascular adverse events, such as cardiac arrest (CA) and ACS. Anecdotal evidence suggests that this might not be only a theoretical concern^16^.

Third, myocardial injury and myocarditis is prevalent among patients with COVID-19 infection^26,34^. As COVID-19 vaccine rollouts often take place with background community COVID-19 infections, it could be challenging to identify whether increased incidence of myocarditis and related cardiovascular conditions, such as CA and ACS, is driven by COVID-19 infections or induced by COVID-19 vaccines. Moreover, such increases may even be caused by other underlying causal mechanisms indirectly related to COVID-19, for example, patients delaying seeking emergent care because of fear of the pandemic and lockdowns^35^.

This study aims to explore how additional data sources, such as those from emergency medical services (EMS), can complement self-reporting vaccine surveillance systems in identifying COVID-19 related public health trends. More specially, the study examines the association between CA and ACS incidents in the 16–39-year-old population and two potential causal factors: COVID-19 infection rates and COVID-19 vaccine rollout. The study leverages the Israel National EMS (IEMS) data system and analyzes all calls related to CA and ACS events over two and a half years, from January 1st, 2019, throughout June 20th, 2021.

## Methods

### Study design

This retrospective population-based study leverages the IEMS data system and analyzes all calls related to CA and ACS events over two and a half years, from January 1st, 2019, to June 20th, 2021. The IEMS call data are coupled with data on COVID-19 infection rates, as well as the respective vaccination rates over the same period of time.

The study’s time period spans 14 months of a ‘normal period’ prior to the COVID-19 pandemic and vaccine rollout (1/2019–2/2020), about 10 months of a ‘pandemic period’ with two waves of the pandemic (3/2020–12/2020), and about 6 months of a ‘pandemic and vaccination period’ (1/2021–6/2021), during which Israel launched its vaccination rollout parallel to a third wave of the COVID-19 pandemic. Thus, it allows to study how CA and ACS call counts change over time with different background conditions and potentially highlight factors that are associated with the observed temporal changes.

This study was deemed exempt from review by the Massachusetts Institute of Technology Institutional Review Board (E-3300). The study was also approved by the research committee of the IEMS.

### Data sources and study population

#### CA and ACS call data

The IEMS data system includes records of all the calls received through Israel’s national emergency telephone number (1-0-1). Note that the IEMS is a national organization that manages all EMS calls in Israel. Each record contains multiple fields of information, including the retrospective verified call-type as determined by the EMS team (as opposed to the initial call classification), date, relevant response characteristics (e.g., death on scene and whether resuscitation was required during the response), and the patient’s age and gender.

The study’s dataset includes all non-cancelled calls with reported patient age and a verified call-type of either CA or ACS. CA calls were defined as a sudden electrical malfunction of the heart of presumed cardiac or medical etiology, resulting in collapse of a patient, excluding CAs related to trauma, drug overdose, or suicide. ACS calls were defined as conditions where the patients experience a reduction in blood flow to the heart that is associated with myocardial infarction.

The call codes used to identify CA and ACS calls are determined by the EMS teams based on defined protocols of the IEMS. CA diagnosis was made based on the circumstances of collapse as described by the caller to the dispatch team, the CA victim’s electrocardiogram (ECG) as obtained through an automated external defibrillator, and common indicators of CA as observed by the responding paramedics (e.g., patient unresponsiveness, agonal breathing). CAs due or obviously related to trauma, drug overdose, or suicide were excluded in this call code and from the study. ACS diagnosis was made based on the patient's 12-lead ECG (a 12-lead ECG was performed on all patients suspected of ACS to confirm the diagnosis), symptoms (e.g., chest pain, shortness of breath), medical history, and physical examination, as obtained by the responding paramedics. Importantly, these protocol and diagnoses were the same throughout the entire study period (2019–2021), allowing for a consistent comparison between the call counts during the baseline, pandemic, and vaccination periods.

The Supplemental Methods describe the IEMS call data fields and call type codes in further detail.

#### Vaccination and COVID-19 infection cases

Data on the vaccinations and COVID-19 cases were obtained from the online Israel Government Database Portal (https://info.data.gov.il/datagov/home/). These data include the number of daily administered 1st and 2nd vaccination doses by age group^36^, as well as the weekly number of new confirmed COVID-19 cases by age group, across all of Israel^37^. The age groups consist of bins of 20 years starting with 0–19. Population counts by similar age groups were also collected from publicly accessible data used to complement these datasets^38^. Note that Israel administered only BNT162b2 vaccines for which the lag between the 1st and 2nd dose is three weeks, and that during January–May 2021, the vaccines were administered to individuals of age 16 and over.

### Data and statistical analyses

#### Trends in CA and ACS calls

For each pair of a diagnosis (CA or ACS), age group (16–39, over-40 or all-ages), and gender (male, female, or both genders) the year-to-year absolute and relative changes in calls were calculated. The respective statistical significance of these changes were based on the two-tailed Poisson E-test^39^. These changes were calculated separately with respect to the full calendar year (2019–2020) and from January 1st to May 31st (2019–2021). January–May time period was used for comparison as it corresponds with the administration of vaccinations among the 16–39 age group in 2021^36^. The full calendar year comparisons were calculated to examine the changes in calls when COVID-19 infections were prevalent, but no vaccinations were administered among the 16–39 age group. Additional analyses describing the percent of CA calls where the patient died on scene (i.e., death declared prior to hospital arrival) and received resuscitation (i.e., patient received defibrillation or cardiopulmonary resuscitation) are outlined in the Appendix.

To visualize the temporal trends of CA and ACS call volume and potential relationship to COVID-19 infection rates and vaccination rates for the 16–39 age group, graphs were created for CA and ACS calls, respectively. Each graph overlays several moving-average time-series over the study period. These include the five-week centered moving-average of the respective weekly EMS call counts, as well as the three-week centered moving-average counts of new COVID-19 infection cases, administered 1st vaccine doses, and administered 2nd vaccine doses. The graphs also indicate the periods of the three national COVID-19-related public health lockdown advisories in Israel^40^.

To improve the understanding these trends during the third pandemic wave and vaccination rollout, ‘zoom-in’ graphs were similarly created for the time-period October 18th, 2020, through June 20th, 2021. The zoom-in graphs also highlight estimates of the number of individuals who only received one vaccination dose during this time. This was done by plotting an additional time-series of the three-week moving-average of the administered 2nd vaccine doses shifted backwards in time by three weeks. More precisely, the difference between the number of 1st vaccine doses and the number of 2nd vaccine doses shifted backwards in time by three weeks shows the estimated number of patients that only received their 1st dose following Pzifer’s vaccination administration recommendations (i.e., the estimated number of patients who did not received a 2nd vaccine dose after a 3-week period following 1st vaccine dose administration). This difference is also used to estimated number of single doses administered to individuals who had recovered from COVID-19 infections, which was plotted from April 1st, 2021, onwards (April 1st 2021 was shortly after the Israel Ministry of Health approved vaccination for this population^41^).

Graphs for the above-40 and all-ages groups are shown in Supplemental Figs. 1–4.

#### Time-series data processing for CA and ACS call, vaccination administration, and COVID-19 infection counts

To check whether the observed year-to-year trends in weekly counts of CA and ACS calls among the 16–39 age group are associated with either COVID-19 infections or vaccine administration, the following weekly time-series were calculated and considered over the entire study period: CA weekly call counts, respectively, for patients in age groups 16–39 and over-40; ACS weekly call counts of patients in age group 16–39; bi-weekly (current and prior week) cumulative counts of 1st and 2nd vaccine doses administered, respectively, in age groups 16–39 and over-40; and cumulative three-week (current and prior two weeks) new COVID-19 infection counts in age groups 16–39 (approximated by age group 0–39) and over-40, respectively. Note that the COVID-19 infection dataset^37^ only includes aggregated data for the age grouping 0–39 and thus overestimates the number of COVID-19 infections for the age group 16–39.

The choice of bi-weekly counts of 1st and 2nd vaccine doses is motivated by studies that suggest myocarditis typically appears within two weeks from vaccination^19^. The choice of three-week cumulative counts of new COVID-19 infections is motivated by the fact that acute symptoms of COVID-19 are typically observed within three weeks of infection onset^19^. Since the impact of COVID-19 might be variable, some of the analysis described below was conducted also with different COVID-19 new infection counts varying the counting period from one to six weeks (i.e., cumulative counts between one, two, three, four, five and six weeks).

#### Association of year-to-year call count trends with COVID-19 infections and vaccine administration

The Spearman rank correlation was calculated between the time-series of CA weekly call counts for the age group 16–39 and the time-series of the bi-weekly (current and prior week) cumulative counts of 1st and 2nd vaccine doses administered for the same age group. Similarly, the rank correlation was calculated between the time-series of the CA weekly call counts and the time series of the cumulative three-week (current and prior two weeks) new COVID-19 infection counts. The same was calculated for the sum of the time-series of CA and ACS weekly call counts for the 16–39 age group (i.e., correlation with the respective time-series of vaccine dose and new COVID-19 infection counts). As mentioned previously, the bi-weekly and three-week cumulative counts for the vaccinations and COVID-19 infections, respectively, were determined based on prior literature suggesting adverse events occur within those respective durations of time^19^. A post hoc power analysis was also performed using G*Power (version 3.1.9.7)^42^ to determine the statistical power (i.e., the probability of rejecting the null hypothesis, concluding an effect is found, and avoiding a Type II error, when an effect truly exists) of the correlation analyses. Finally, since the impact of COVID-19 might occur across a variable period of time, the same analysis was repeated with respect to the time-series of new COVID-19 infections count but varying the cumulative count period from the original three-weeks to a range between one to six weeks.

To further study the potential association between weekly CA and ACS counts, vaccine administration and COVID-19 infections, and control for cross interactions and other factors, two Negative Binomial regression models^43^ were developed. Negative binomial regression models are commonly used to model count data and allows for the analysis of cases where the outcome variable counts are over-dispersed (variance of the count data is larger than the mean)^43,44^. Such models can also be designed to use cumulative historical count data as features to estimate outcome counts during a given current time period^35,45,46^.

The first model, hereinafter referred to as *Model 1*, regresses the respective time-series of the CA weekly call counts and the ACS weekly call counts in the age group 16–39 (the dependent variable), against the time-series of the bi-weekly cumulative vaccine dose counts and three-week cumulative new COVID-19 infection counts, both in age group 16–39 normalized by the respective population size (independent variables). The model also controls for the different diagnoses (CA versus ACS), for weeks included in periods of national public health lockdown, as well as year-to-year (2019–2020) variations (e.g., due to population growth) in calls through respective dummy variables.

Similarly, the second model, hereinafter referred to as *Model 2*, regresses the respective time-series of CA weekly counts of age groups 16–39 and over-40 (the dependent variable) against the time-series of the bi-weekly cumulative vaccine dose counts and three-week cumulative new COVID-19 infection counts in the respective age groups, again normalized by the respective population size (independent variables). Additionally, instead of the dummy variable used in Model 1 above to capture the different diagnosis groups, Model 2 introduces a dummy variable to capture the different age groups (16–39 and over-40).

To identify the most statistically significant predictors, the models use bidirectional stepwise feature selection based on the model’s Bayesian information criterion (BIC). The BIC metric summarizes the model’s goodness of fit while penalizing the number of variables selected to avoid overfitting^47^. During each step of the selection algorithm, features are tested to be added or removed to minimize the model’s BIC. The adjusted incidence rate ratios (IRR) and 95% confidence intervals (CI), representing the estimated change in weekly calls per unit change of each predictor variable, were reported both for the final model after stepwise BIC selection and the full model without variable selection. Model development was performed using R version 4.0.2.

##### Sensitivity analysis

As robustness check of the associations determined by Models 1 and 2, the analysis was repeated while considering the one to six-week count time-series of new COVID-19 infections in the respective age groups.

### Patient and public involvement

The formal involvement of the public and patients was not feasible under the time and resources constraints of this research project. However, this work has been informed by dialogue with those working in healthcare systems and public policy.

### Ethical approval

This study was deemed exempt from review by the Massachusetts Institute of Technology Institutional Review Board(E-3300). The study was also approved by the research committee of the IEMS.

## Results

### General descriptive results

Of the 30,481 cardiac arrest and 61,001 ACS calls included in the study population (see Supplemental Results for details), 952 (3.1%) and 3,994 (6.5%) calls were for patients of age 16–39, respectively, from a population of close to 3.5 million people in this age group^38^. Of the 834,573 confirmed COVID-19 cases during the study period, 572,435 (68.6%) cases were from individuals of age 16–39. Among the 5,506,398 patients receiving their 1st vaccination dose and 5,152,417 patients receiving their 2nd vaccination dose, 2,382,864 (43.3%) and 2,176,172 (42.2%) patients were of age 16–39, respectively.

### Year-to-year changes in CA and ACS calls

Table 1 summarizes the year-to-year changes in CA and ACS call volume. The results highlight a statistically significant increase of 25% in both CA (25.7%, P < 0.05) and ACS (24.6%, P < 0.001) calls for patients of ages 16–39 during January–May 2021, compared to the same period in 2020. Interestingly, for CA, there is no statistically significant difference in the respective call volume across the full year (January–December) from 2019 to 2020 (relative increase of 0.5% [P = 0.942]), prior to the vaccination rollout and third COVID-19 wave in this age group. Similarly, for ACS, the increase across the full year from 2019 and 2020 (significant relative increase of 15.8% [P < 0.001]) was followed by an even larger increase in the January to May period from 2020 to 2021 (significant relative increase of 24.6% [P < 0.001]), which was during the third COVID-19 wave and vaccination rollout. Both genders in the 16–39 age group experienced increases in CA and ACS calls from 2020 to 2021 for January–May. Among males, CA calls increased by 27.0% (P = 0.055) and ACS calls increased significantly by 20.4% (P < 0.01). Among females, CA calls increased by 25.0% (P = 0.321) and ACS calls instead significantly by 37.7% (P < 0.01).

**Table 1:** Year-to-year absolute and relative changes in the counts of cardiac arrest and acute coronary syndrome calls by age group and gender.

Gender: age group	Cardiac arrest, Counts (Percent change relative to previous year; P-value)					Acute coronary syndrome, Counts (Percent change relative to previous year; P-value)				
	Full year counts		January–May counts			Full year counts		January–May counts		
	2019	2020 (Percent change relative to 2019; P-value)	2019	2020 (Percent change relative to January–May 2019; P-value)	2021 (Percent change relative to January–May 2020; P-value)	2019	2020 (Percent change relative to 2019; P-value)	2019	2020 (Percent change relative to January–May 2019; P-value)	2021 (Percent change relative to January–May 2020; P-value)
All: overall*	11,187 (–)	12,866 (15.0; P < 0.001)	4994 (–)	5379 (7.7; P < 0.001)	5651 (5.1; P < 0.01)	23,313 (–)	24,501 (5.1; P < 0.001)	9293 (–)	9762 (5.1; P < 0.001)	11,204 (14.8; P < 0.001)
All: 16–39*	367 (–)	369 (0.5; P = 0.942)	136 (–)	152 (11.8; P = 0.347)	191 (25.7; P < 0.05)	1418 (–)	1642 (15.8; P < 0.001)	548 (–)	635 (15.9; P < 0.05)	791 (24.6; P < 0.001)
All: over 40*	10,820 (–)	12,497 (15.5; P < 0.001)	4858 (–)	5227 (7.6; P < 0.001)	5460 (4.5; P < 0.05)	21,895 (–)	22,859 (4.4; P < 0.001)	8745 (–)	9127 (4.4; P < 0.01)	10,413 (14.1; P < 0.001)
Female: overall	5496 (–)	6282 (14.3; P < 0.001)	2510 (–)	2644 (5.3; P = 0.062)	2759 (4.4; P = 0.118)	7904 (–)	8768 (10.9; P < 0.001)	3167 (–)	3483 (10.0; P < 0.001)	4125 (18.4; P < 0.001)
Female: 16–39	104 (–)	87 (–16.4; P = 0.220)	34 (–)	36 (5.9; P = 0.818)	45 (25.0; P = 0.321)	305 (–)	411 (34.8; P < 0.001)	112 (–)	154 (37.5; P < 0.01)	212 (37.7; P < 0.01)
Female: over 40	5392 (–)	6195 (14.9; P < 0.001)	2476 (–)	2608 (5.3; P = 0.064)	2714 (4.1; P = 0.146)	7599 (–)	8357 (10.0; P < 0.001)	3055 (–)	3329 (9.0; P < 0.001)	3913 (17.5; P < 0.001)
Male: overall	5670 (–)	6583 (16.1; P < 0.001)	2475 (–)	2734 (10.5; P < 0.001)	2892 (5.8; P < 0.05)	15,307 (–)	15,732 (2.8; P < 0.05)	6065 (–)	6279 (3.5; P = 0.054)	7079 (12.7; P < 0.001)
Male: 16–39	260 (–)	281 (8.1; P = 0.367)	101 (–)	115 (13.9; P = 0.342)	146 (27.0; P = 0.055)	1107 (–)	1231 (11.2; P < 0.05)	433 (–)	481 (11.1; P = 0.112)	579 (20.4; P < 0.01)
Male: over 40	5410 (–)	6302 (16.5; P < 0.001)	2374 (–)	2619 (10.3; P < 0.001)	2746 (4.9; P = 0.083)	14,200 (–)	14,501 (2.1; P = 0.076)	5632 (–)	5798 (3.0; P = 0.121)	6500 (12.1; P < 0.001)

Each cell shows the counts of calls during the respective time period, age group, and gender with the relative percent change in counts to the previous year shown in the parenthesis (e.g., relative change from 2019 to 2020, and then from 2020 to 2021). The relative percent changes were calculated across the same duration per year (i.e., either across the full year or across the January–May period). For counts during 2019, no relative change is reported.

*Counts in the All category includes calls with missing gender variable values. Number of calls with missing gender values: Cardiac arrest: N = 119 and Acute Coronary syndrome: N = 183.

Supplemental Table 1 shows the year-to-year percent of CA patients who died on scene (i.e., prior to hospital arrival) for the same time periods. Among the 16–39 age group, the percent of CA patients that died prior to hospital arrival increased significantly from 2019 to 2020 during the full year (50.7–59.9%; P < 0.05). This percentage remained elevated during January–May of 2021 and no significant differences were found between the same period in 2020 (65.1–58.1% P = 0.185). Similarly, Supplemental Table 2 shows that in the 16–39 age group, resuscitation (i.e., the patient received defibrillation or cardiopulmonary resuscitation delivery) rates for CA calls increased from 2019 to 2020 during the full year (43.9–55.8%; P < 0.01). These higher rates of resuscitation persisted during January–May 2021, with no significant difference compared to the same period in 2020 (55.9–56.5%; P = 0.91).

### Association between CA and ACS calls to COVID-19 infections and vaccine administration

Considering the age group 16–39, the Spearman rank correlation between the CA weekly call counts and the cumulative bi-weekly (current and prior week) 1st and 2nd doses count is 0.209 (P < 0.05). The correlation factor of the sum of the weekly CA and ACS call counts with the same vaccine count time-series is 0.457 (P < 0.001). The post hoc power analysis found that the statistical power for a significance level of 0.05 were 0.67 and 0.9999 for the correlation between vaccination doses and CA weekly call counts, and the sum of CA and ACS weekly call counts, respectively. The time-series of the cumulative three-week (current and two prior weeks) new COVID-19 infections count was not significantly correlated with the CA weekly call count time-series (0.076, P = 0.392), while the time-series sum of CA and ACS weekly call counts was significantly correlated (0.410, P < 0.001). The post hoc power analysis found that the statistical power for a significance level of 0.05 was 0.14 and 0.9990 for the correlation between COVID-19 infection and CA weekly call counts, and the sum of CA and ACS weekly call counts, respectively. The same patterns hold when the COVID-19 infection count period is varied between one to six weeks (Supplemental Table 3).

**Figure 1:**
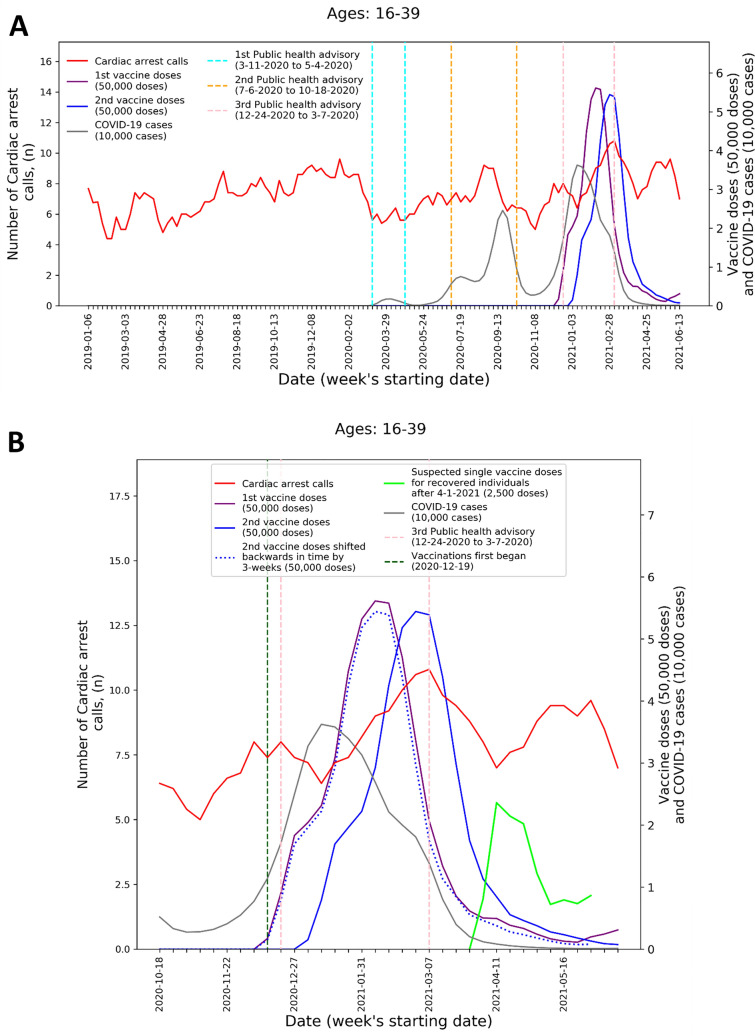
Weekly counts of cardiac arrest calls (five-week centered moving-average), COVID-19 cases (three-week centered moving-average), and vaccination doses (three-week centered moving-average) for those between 16 and 39 during: A) the study period (January 1st, 2019, to June 20th, 2021) and B) the third COVID-19 wave and vaccination distribution period (October 18th, 2020, to June 20th, 2021). *COVID-19* Coronavirus disease 2019.

**Figure 2:**
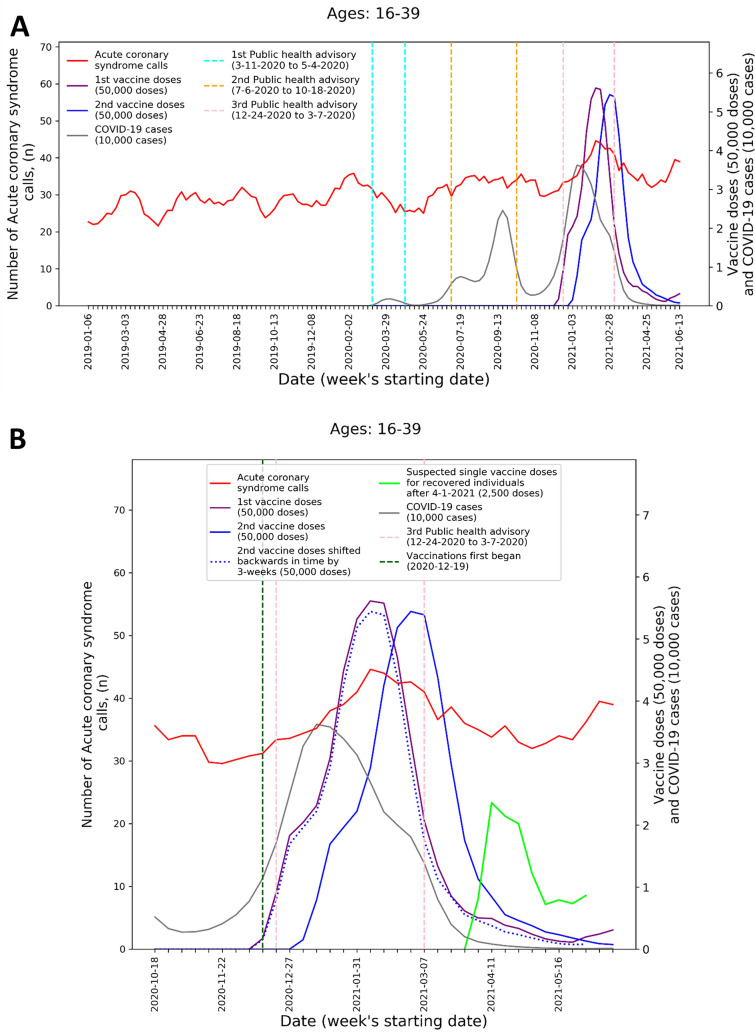
Weekly counts of acute coronary syndrome calls (five-week centered moving-average), COVID-19 cases (three-week centered moving-average), and vaccination doses (three-week centered moving-average) for those between 16 and 39 during: A) the study period (January 1st, 2019, to June 20th, 2021) and B) the third COVID-19 wave and vaccination distribution period (October 18th, 2020, to June 20th, 2021). *COVID-19* Coronavirus disease 2019.

These findings are emphasized by Figs. 1 and 2 that present the graphs described in the “Methods” section for both CA and ACS, CA only, and ACS only, respectively. Both the CA and ACS call counts (red curve) start increasing early January 2021 and seem to track closely the 2nd dose curve (solid blue curve). They peak around early March and then decrease during March and the first part of April (Figs. 1B and 2B). The graphs also highlight the lack of association between the COVID-19 infection counts (grey curve) and the CA and ACS call counts, which is most clearly seen during the first two major infection waves in 2020.

A second increase is observed starting around April 18th. Interestingly, this second increase seems to track closely the estimated number of single doses delivered for individuals who recovered from COVID-19 (green line), starting on April 11th. In early March the Israel Ministry of Health approved the vaccination of individuals of age 16 and over, who recovered from a COVID-19 infection, with only one vaccine dose, as long as three months elapsed from their recovery^41^. As can be seen from the COVID-19 infection counts, the peak of the third wave among people under 40 occurred around January 11th. This could explain the potential increase in one-dose vaccination observed starting April 11th.

### Negative binomial regression models results

Table 2 below shows the results for Model 1 described in the “Methods” section (the dependent variable: time-series of CA weekly call counts the ACS weekly call counts, both in age group 16–39). With BIC feature selection, the bi-weekly cumulative counts of 1st and 2nd vaccine doses in the age group 16–39 (normalized by the respective population size), was selected as statistically significant predictor with a positive relationship to the dependent variables (IRR: 3.32, [95% CI 2.12–5.14]). That is, increased rates of vaccination in the respective age group are associated with increased number of CA and ACS weekly call counts. In contrast, the three-week cumulative new COVID-19 infection counts among the age group 16–39 (normalized by the respective population size) was not selected as a predictor of the call counts time-series. That is, the model did not detect a statistically significant association between the COVID-19 infection rates and the CA and ACS weekly call counts.

**Table 2:** Associations with cardiac arrest and acute coronary syndrome calls among those aged 16–39 using a negative binomial regression model, with and without stepwise BIC feature selection.

Variable	With stepwise BIC selection		Without feature selection	
	Adjusted incidence rate ratio (95% CI)	P-value	Adjusted incidence rate ratio (95% CI)	P-value
The bi-weekly cumulative counts of 1st and 2nd vaccine doses in the age group 16–39, normalized by the respective population size	3.32 (2.12–5.14)	< 0.001	2.20 (1.06–4.56)	< 0.05
The three-week cumulative new COVID-19 infection count among the age group 16–39, normalized by the respective population size	–	–	26.94 (0.03–20,067.17)	0.330
Call type: Acute coronary syndrome	1 [Reference]	–	1 [Reference]	–
Call type: Cardiac arrest	0.24 (0.22–0.25)	< 0.001	0.24 (0.22–0.25)	< 0.001
Week not during a COVID-19 public health advisory	1 [Reference]	–	1 [Reference]	–
Week during a COVID-19 public health advisory	–	–	0.94 (0.85–1.04)	0.224
Year: 2019	0.88 (0.83–0.94)	< 0.001	0.83 (0.75–0.92)	< 0.001
Year: 2020	–	–	0.94 (0.84–1.05)	0.245
Year: 2021	1 [Reference]	–	1 [Reference]	–

*BIC* Bayesian information criterion, *CI* confidence interval, *COVID-19* coronavirus disease 2019.

Similar results are obtained without feature selection. The time-series of vaccine dose counts still had a statistically significant positive relationship with the CA and ACS weekly call counts (IRR: 2.20, [95% CI 1.06–4.56]), while the time-series of new COVID-19 infection counts did not have statistical significance. Additionally, national public health lockdown periods did not have statistical significance. The adjusted R^2^ was 0.872 and 0.874 with and without feature selection, respectively.

Table 3 shows the results for Model 2 described in the “Methods” section (the dependent variable: the time-series of CA weekly call counts of the respective age groups 16–39 and over-40). Like in the analysis of Model 1 above, with the BIC feature selection, the time-series of vaccine doses were selected as statistically significant with positive associated with the dependent variable of CA weekly call counts (IRR: 1.79, 95% CI [1.43–2.23]), whereas the time-series of the new COVID-19 infection counts was not selected. Without feature selection, the time-series of vaccine dose counts remained statistically significant and positive (IRR: 1.94, 95% CI [1.36–2.76]) and the time-series of new COVID-19 infection counts did not have statistical significance. The national public health lockdown periods were also not statistically significant. The adjusted R^2^ was 0.937 and 0.938 for the with and without feature selection models, respectively.

**Table 3:** Associations with cardiac arrest calls among all ages using a negative binomial regression model, with and without stepwise BIC feature selection.

Variable	With stepwise BIC selection		Without feature selection	
	Adjusted incidence rate ratio (95% CI)	P-value	Adjusted incidence rate ratio (95% CI)	P-value
The bi-weekly cumulative counts of 1st and 2nd vaccine doses per age group, normalized by the respective population size	1.79 (1.43–2.23)	< 0.001	1.94 (1.36–2.76)	< 0.001
The three-week cumulative new COVID-19 infection count per age group, normalized by the respective population size	–	–	5.49 (0.001–19,001.98)	0.685
Age group: Below 40	1 [Reference]	–	1 [Reference]	–
Age group: 40 and above	31.08 (29.01–33.33)	< 0.001	31.16 (29.01–33.52)	< 0.001
Week not during a COVID-19 public health advisory	1 [Reference]	–	1 [Reference]	–
Week during a COVID-19 public health advisory	–	–	0.98 (0.92–1.05)	0.630
Year: 2019	0.89 (0.86–0.93)	< 0.001	0.92 (0.86–0.99)	< 0.05
Year: 2020	–	–	1.05 (0.98–1.12)	0.178
Year: 2021	1 [Reference]	–	1 [Reference]	–

*BIC* Bayesian information criterion, *CI* confidence interval, *COVID-19* coronavirus disease 2019.

#### Sensitivity analysis

For each model, the new COVID-19 infection normalized counts time-series is never selected as a significant variable, even when the count period is varied between one to six weeks. At the same time the vaccine doses normalized counts time-series is always selected as a statistically significant variable with positive association (see Supplemental Tables 4–7).

## Discussion

This study leverages a unique dataset of all EMS CA and ACS calls in Israel over two and half years that span 14 months prior to the start of the COVID-19 pandemic, 10 months that include two waves of the COVID-19 pandemic, and 6 months with a third wave of the pandemic parallel to the vaccination rollout among the 16-year-old and over population. Thus, it provides a unique perspective to explore the association between trends in CA and ACS call volume over the study period and different factors, such as COVID-19 infection rates and vaccination rates.

Moreover, because the IEMS is a national organization the data provide a more comprehensive access to the respective incidence of the conditions being studied. This stands in contrast to the known very partial and biased access provided by adverse event self-reporting surveillance systems^23–25^, and highlights the importance of incorporating additional data sources into these systems^48^. However, it is important to highlight several significant differences between the CA and ACS EMS calls. For CA events, it is reasonable to assume that the IEMS data includes almost all of the relevant events, since CA events almost always involve calling EMS services. Moreover, the diagnosis of CA is relatively more straightforward. In contrast, for ACS events, while EMS calls capture a significant fraction of the respective incidents, direct hospital walk-in will not be accounted for in the EMS data. In Israel this is estimated to be 50% of all events. Additionally, the diagnosis of ACS events is more involved, and while EMS protocols during the study period did not change, it is reasonable to assume a higher rate of diagnosis error.

The main finding of this study concerns with increases of 25% in both the number of CA calls and ACS calls of people in the 16–39 age group during the COVID-19 vaccination rollout in Israel (January–May, 2021), compared with the same period of time in prior years (2019 and 2020), as shown in Table 1. Moreover, there is a robust and statistically significant association between the weekly CA and ACS call counts, and the rates of 1st and 2nd vaccine doses administered to this age group. At the same time there is no observed statistically significant association between COVID-19 infection rates and the CA and ACS call counts. This result is aligned with previous findings which show increases in overall CA incidence were not always associated with higher COVID-19 infections rates at a population level^35,49,50^, as well as the stability of hospitalization rates related to myocardial infarction throughout the initial COVID-19 wave compared to pre-pandemic baselines in Israel^51^. These results also are mirrored by a report of increased emergency department visits with cardiovascular complaints during the vaccination rollout in Germany^52^ as well as increased EMS calls for cardiac incidents in Scotland^53^.

The visuals in Figs. 1 and 2 support and reinforce these findings. The increase in CA and ACS calls starting early January 2021 seems to track closely the administration of 2nd dose vaccines. This observation is consistent with prior findings that associated more significant adverse events, including myocarditis to the 2nd dose of the vaccine^19^. A second increase in the CA and ACS call counts is observed starting April 18th, 2021, which seems to track an increase of single-dose vaccination to individuals who recovered from COVID-19 infections. This is consistent with prior findings that suggest that the immune response generated by a single dose on recovered individuals is generally stronger than the response to the 2nd vaccine dose in individuals, who were not exposed to COVID-19 infection^54^. Additionally, the graphs emphasize the absence of correlation between the call counts and COVID-19 infection counts, which is most clearly seen during the two major pandemic waves in 2020.

While increased CA incidence was not observed among the 16–39 age group in 2020, there was a significant increase in the proportion of CA patients that died on scene during 2020, relative to 2019 (Supplemental Table 1), emphasizing the potential direct and indirect harmful effects of the pandemic^35,49,55^ on out-of-hospital CA patient outcomes. The percent of patients that died on scene remained elevated in 2021.

The large increase in the incidence of CA and ACS events in the population of age 16–39 parallel to the vaccination rollout and its association with the vaccination rates could be consistent with the known causal relationship between the mRNA vaccines and incidents of myocarditis in young people^14,17,19,56^, as well as the fact that myocarditis is often misdiagnosed as ACS^28–30^, and that asymptomatic myocarditis is a frequent cause for unexplained sudden death among young adults from CA^26,31–33^. This is further supported by more anecdotal reports describing sudden cardiac death following COVID-19 vaccination^16,57^. While vaccine-induced myocarditis was predominantly reported in males^14,19^ it is interesting to note that the relative increases of CA and ACS events (Table 1) was larger in females. This may suggest the potential underdiagnosis or under-self-reporting of myocarditis in females, or other unique patterns, which is consistent with the ongoing challenge of gender-related differences related to cardiovascular disease diagnosis and care^15,58^.

The paper suggests several important policy implications. First, it is important that surveillance programs of potential vaccine side-effects and COVID-19 infection outcomes incorporate EMS and other health data to identify public health trends and promptly investigate potential underlying causes. Specifically, prompt investigation is needed to better understand the potential underlying causes of the observed increase in cardiac-related EMS calls, including vaccine and COVID-19 infection related factors, as well as additional factors, such as reduced willingness to seek hospital or EMS care, reduced access to care, and increased public awareness to post-vaccination adverse events. Second, it is essential to raise awareness among patients and clinicians with respect to related symptoms (e.g., chest discomfort and shortness of breath) following vaccination or COVID-19 infection to ensure that potential harm is minimized. This is especially important among the younger population and particularly young females, who often receive less diagnostic evaluation for adverse cardiac events compared to males^15^. These implications are further underscored by the continued administration of additional vaccine booster doses to the public because of the waning vaccine immunity against COVID-19 variants (e.g., delta variant) after the 2nd vaccine dose^59^. Moreover, recent studies have also demonstrated the association of increased risk of myocarditis with the administration of adenovirus-based vaccines (i.e., ChAdOx1)^17^, in addition to mRNA vaccinations, increasing the number of individuals that could be susceptible potential vaccine side-effects as well that can benefit from enhanced vaccine surveillance programs.

It is important to note the main limitation of this study, which is that it relies on aggregated data that do not include specific information regarding the affected patients, including hospital outcomes, underlying comorbidities as well as vaccination and COVID-19 positive status. Such related data are critical to determine the exact nature of the observed increase in CA and ACS calls in young people, and what the underlying causal factors are. Notably, recent studies have found vaccination induced myocardial injury has differentiating features, such as histopathology^60^, compared to typical myocarditis, which can further support identification of possible drivers of these cardiac events. The Israel Ministry of Health and the large HMOs have access to such data, which should be investigated in detail. Additionally, the CA examined in the study included those of both cardiac and medical etiology as data discerning these differences were not available, increasing the importance of further investigation of these patients. However, previous literature has estimated that the vast majority, approximately 84–92%, of non-traumatic cardiac arrest cases stem from cardiac origins^61^. For example, among other potential causes of CA, approximately 2–9% and 2% of cardiac arrests stem from pulmonary embolism^62,63^ and acute cerebrovascular events (e.g. subarachnoid hemorrhage)^64^, respectively. Therefore, it is likely that the observed changes in incidence can primarily be attributed to CAs of cardiac etiology.

The significant increases in CA calls and ACS calls among the 16–39 age population during the COVID-19 vaccination rollout highlights the value of additional data sources, such as those from EMS systems, that can supplement self-reporting surveillance systems in identifying concerning public health trends. Moreover, it underscores the need for the thorough investigation of the apparent association between COVID-19 vaccine administration and adverse cardiovascular outcomes among young adults. Israel and other countries should immediately collect the data necessary to determine whether such association indeed exists, including thorough investigation of individual CA and ACS cases in young adults, and their potential connection to the vaccine or other factors. This would be critical to better understanding the risk-benefits of the vaccine and to inform related public policy and prevent potentially avoidable patient harm. In the interim, it is vital that following vaccination, patients should be instructed to seek appropriate emergency care if they are experiencing symptoms potentially associated with myocarditis, such as chest discomfort and shortness of breath, as well as consider avoiding strenuous physical activity following the vaccination that may induce severe adverse cardiac events.

## Supplementary Information

Supplementary Information.

## Data Availability

The COVID-19 and vaccination rate datasets generated and analysed during the current study are available at https://data.gov.il/dataset/covid-19. EMS call count data are not publicly available as they are derived from national clinical records. Due to national and organizational data privacy regulations this data cannot be shared openly. All the data files, templates, codes and respective output files used in the analyses are deposited on Figshare and can be accessed at 10.6084/m9.figshare.23805954.v1.
