# Author Correction: Increased emergency cardiovascular events among under-40 population in Israel during vaccine rollout and third COVID-19 wave

**DOI:** 10.1038/s41598-023-40234-1

**Published:** 2023-08-15

**Authors:** Christopher L. F. Sun, Eli Jaffe, Retsef Levi

**Affiliations:** 1 Sloan School of Management, Massachusetts Institute of Technology, 100 Main Street, Cambridge, MA 02142‑1347 USA; 2 Healthcare Systems Engineering, Massachusetts General Hospital, Boston, MA USA; 3 Israel National Emergency Medical Services (Magen David Adom), Tel Aviv‑Jaffo, Israel; 4 Ben Gurion University of the Negev, Beer Sheva, Israel

Correction to: *Scientific Reports* 10.1038/s41598-022-10928-z, published online 28 April 2022

The original version of this Article contained errors.

First, in calculating the Spearman rank correlations between the time-series of weekly administered vaccine doses and COVID-19 infections, respectively, with the sum of the weekly emergency medical service (EMS) CA and ACS call counts, we discovered a coding error in calculating the latter time series. Specifically, the application of the incorrect code function led to concatenating the two time-series (CA and ACS weekly calls) instead of summing them. This was accompanied by an error in the corresponding post hoc power analysis, specifically, applying the post-hoc power software with the wrong (larger) input sample size parameters resulting in incorrect higher post-hoc power values. Correcting these errors led to higher Spearman correlation factors and lower p-values, as well as corrected post-hoc power values. Finally, we found an error in how data were extracted for a small fraction of EMS calls with multiple records as a result of multiple ambulances called to the scene. This correction led to a more accurate determination of the age range as well as the diagnosis of these calls, and resulted in very small changes in the number of weekly EMS calls included in the analyses, as well as in the corresponding Tables and Figures.

As a result, in the Abstract,

“An increase of over 25% was detected in both call types during January–May 2021, compared with the years 2019–2020”

now reads:

“An increase of 25% was detected in both call types during January–May 2021, compared with the years 2019–2020”

In the Results section, subheading ‘General descriptive results’,

“Of the 30262 cardiac arrest and 60398 ACS calls included in the study population (see Supplementary Results for details), 945 (3.1%) and 3945 (6.5%) calls were for patients of age 16–39, respectively”

now reads:

“Of the 30481 cardiac arrest and 61001 ACS calls included in the study population (see Supplementary Results for details), 952 (3.1%) and 3994 (6.5%) calls were for patients of age 16–39, respectively”

In the Results section, subheading ‘Year-to-year changes in CA and ACS calls’,

“Table 1 summarizes the year-to-year changes in CA and ACS call volume. The results highlight a statistically significant increase of over 25% in both CA (25.7%, P<0.05) and ACS (26.0%, P<0.001) calls for patients of ages 16–39 during January-May 2021, compared to the same period in 2020. Interestingly, for CA, there is no statistically significant difference in the respective call volume across the full year (January-December) from 2019 to 2020 (relative decrease of −2.4% [P=0.740]), prior to the vaccination rollout and third COVID-19 wave in this age group. Similarly, for ACS, the increase across the full year from 2019 and 2020 (significant relative increase of 15.8% [P<0.001]) was followed by an even a larger increase in the January to May period from 2020 to 2021 (significant relative increase of 26.0% [P<0.001]), which was during the third COVID-19 wave and vaccination rollout. Both genders in the 16–39 age group experienced increases in CA and ACS calls from 2020 to 2021 for January-May. Among males, CA calls increased by 25.0% (P=0.073) and ACS calls increased significantly by 21.3% (P<0.01). Among females, CA calls increased by 31.4% (P=0.224) and ACS calls instead significantly by 40.8% (P<0.01).”

now reads:

“Table 1 summarizes the year-to-year changes in CA and ACS call volume. The results highlight a statistically significant increase of 25% in both CA (25.7%, P < 0.05) and ACS (24.6%, P < 0.001) calls for patients of ages 16–39 during January–May 2021, compared to the same period in 2020. Interestingly, for CA, there is no statistically significant difference in the respective call volume across the full year (January–December) from 2019 to 2020 (relative increase of 0.5% [P=0.942]), prior to the vaccination rollout and third COVID-19 wave in this age group. Similarly, for ACS, the increase across the full year from 2019 and 2020 (significant relative increase of 15.8% [P < 0.001]) was followed by an even larger increase in the January to May period from 2020 to 2021 (significant relative increase of 24.6% [P < 0.001]), which was during the third COVID-19 wave and vaccination rollout. Both genders in the 16–39 age group experienced increases in CA and ACS calls from 2020 to 2021 for January–May. Among males, CA calls increased by 27.0% (P = 0.055) and ACS calls increased significantly by 20.4% (P < 0.01). Among females, CA calls increased by 25.0% (P = 0.321) and ACS calls instead significantly by 37.7% (P < 0.01).”

And,

“Among the 16–39 age group, the percent of CA patients that died prior to hospital arrival increased significantly from 2019 to 2020 during the full year (52.8% to 60.5%; P<0.001). This percent remained elevated during January-May of 2021 and no significant differences were found between same period in 2020 (65.1% to 61.3% P=0.460). Similarly, Supplemental Table 2 shows that in the 16–39 age group, resuscitation (i.e., patient received defibrillation or cardiopulmonary resuscitation delivery) rates for CA calls increased from 2019 to 2020 during the full year (41.5% to 54.4%; P<0.001). These higher rates of resuscitation persisted during January-May 2021, with no significant difference compared to the same period in 2020 (54.6% to 53.9%; P=0.900).”

now reads:

“Among the 16–39 age group, the percent of CA patients that died prior to hospital arrival increased significantly from 2019 to 2020 during the full year (50.7–59.9%; P < 0.05). This percentage remained elevated during January–May of 2021 and no significant differences were found between the same period in 2020 (65.1–58.1% P = 0.185). Similarly, Supplemental Table 2 shows that in the 16–39 age group, resuscitation (i.e., the patient received defibrillation or cardiopulmonary resuscitation delivery) rates for CA calls increased from 2019 to 2020 during the full year (43.9–55.8%; P < 0.01). These higher rates of resuscitation persisted during January–May 2021, with no significant difference compared to the same period in 2020 (55.9–56.5%; P = 0.91).”

In the Results section, subheading ‘Association between CA and ACS calls to COVID-19 infections and vaccine administration’,

“The correlation factor of the sum of the weekly CA and ACS call counts with the same vaccine count time-series is 0.164 (P < 0.01).”

now reads:

“The correlation factor of the sum of the weekly CA and ACS call counts with the same vaccine count time-series is 0.457 (P < 0.001).”

And,

“In contrast, the time-series of the cumulative three-week (current and two prior weeks) new COVID-19 infections count was not significantly correlated to either the CA weekly call count time-series (0.047, P = 0.600) or the time-series sum of CA and ACS weekly call counts (0.117, P = 0.061), respectively.”

now reads:

“The time-series of the cumulative three-week (current and two prior weeks) new COVID-19 infections count was not significantly correlated with the CA weekly call count time-series (0.076, P = 0.392), while the time-series sum of CA and ACS weekly call counts was significantly correlated (0.410, P < 0.001).”

And,

“The post hoc power analysis found that the statistical power for a significance level of 0.05 were both 1.00 for the correlation between vaccination doses and CA weekly call counts, and sum of CA and ACS weekly call counts, respectively.”

now reads:

“The post hoc power analysis found that the statistical power for a significance level of 0.05 were 0.67 and 0.9999 for the correlation between vaccination doses and CA weekly call counts, and the sum of CA and ACS weekly call counts, respectively.”

And,

“The post hoc power analysis found that the statistical power for a significance level of 0.05 was 0.94 and 1.00 for the correlation between COVID-19 infection and CA weekly call counts, and sum of CA and ACS weekly call counts, respectively.”

now reads:

“The post hoc power analysis found that the statistical power for a significance level of 0.05 was 0.14 and 0.9990 for the correlation between COVID-19 infection and CA weekly call counts, and the sum of CA and ACS weekly call counts, respectively.”

In the Results, subheading ‘Negative binomial regression models results’.

“With BIC feature selection, the bi-weekly cumulative counts of 1st and 2nd vaccine doses in the age group 16–39 (normalized by the respective population size), was selected as statistically significant predictor with a positive relationship to the dependent variables (IRR: 3.33, [95% CI 2.14–5.14]).”

now reads:

“With BIC feature selection, the bi-weekly cumulative counts of 1st and 2nd vaccine doses in the age group 16–39 (normalized by the respective population size), was selected as statistically significant predictor with a positive relationship to the dependent variables (IRR: 3.32, [95% CI 2.12–5.14]).”

And,

“The time-series of vaccine dose counts still had a statistically significant positive relationship with the CA and ACS weekly call counts (IRR: 2.12, [95% CI: 1.05–4.22]), while the time-series of new COVID-19 infection counts did not have statistical significance. Additionally, national public health lockdown periods did not have statistical significance. The adjusted R^2^ was 0.874 and 0.876 with and without feature selection, respectively.”

now reads:

“The time-series of vaccine dose counts still had a statistically significant positive relationship with the CA and ACS weekly call counts (IRR: 2.20, [95% CI 1.06–4.56]), while the time-series of new COVID-19 infection counts did not have statistical significance. Additionally, national public health lockdown periods did not have statistical significance. The adjusted R^2^ was 0.872 and 0.874 with and without feature selection, respectively.”

And,

“Like in the analysis of Model 1 above, with the BIC feature selection, the time-series of vaccine doses was selected as a statistically significant with positive associated with the dependent variable of CA weekly call counts (IRR: 1.79, 95% CI [1.43–2.25]), whereas the time-series of the new COVID-19 infection counts was not selected. Without feature selection, the time-series of vaccine dose counts remained statistically significant and positive (IRR: 1.92, 95% CI [1.34–2.76]) and the time-series of new COVID-19 infection counts did not have statistical significance. The national public health lockdown periods were also not statistically significant. The adjusted R^2^ was 0.930 and 0.932 for the with and without feature selection models, respectively.”

now reads:

“Like in the analysis of Model 1 above, with the BIC feature selection, the time-series of vaccine doses were selected as statistically significant with positive associated with the dependent variable of CA weekly call counts (IRR: 1.79, 95% CI [1.43–2.23]), whereas the time-series of the new COVID-19 infection counts was not selected. Without feature selection, the time-series of vaccine dose counts remained statistically significant and positive (IRR: 1.94, 95% CI [1.36–2.76]) and the time-series of new COVID-19 infection counts did not have statistical significance. The national public health lockdown periods were also not statistically significant. The adjusted R^2^ was 0.937 and 0.938 for the with and without feature selection models, respectively.”

Moreover, in the Discussion section,

“The main finding of this study concerns with increases of over 25% in both the number of CA calls and ACS calls of people in the 16–39 age group during the COVID-19 vaccination rollout in Israel (January–May 2021), compared with the same period of time in prior years (2019 and 2020), as shown in Table 1.”

now reads:

“The main finding of this study concerns with increases of 25% in both the number of CA calls and ACS calls of people in the 16–39 age group during the COVID-19 vaccination rollout in Israel (January–May 2021), compared with the same period of time in prior years (2019 and 2020), as shown in Table 1.”

Data shown in Tables 1–3 and Supplementary Tables 1–7 was updated accordingly, the original Tables are shown below, and the original Supplementary Information file is appended to this notice for reference.

Incorrect Table 1:

**Table:** 

Gender: age group	Cardiac arrest, Counts (Percent change relative to previous year; P-value)					Acute coronary syndrome, Counts (Percent change relative to previous year; P-value)				
	Full year counts		January–May counts			Full-year counts		January–May counts		
	2019	2020 (Percent change relative to 2019; P-value)	2019	2020 (Percent change relative to January–May 2019; P-value)	2021 (Percent change relative to January–May 2020; P-value)	2019	2020 (Percent change relative to 2019; P-value)	2019	2020 (Percent change relative to January–May 2019; P-value)	2021 (Percent change relative to January–May 2020; P-value)
All: overall*	11,149 (–)	12,792 (14.7; P < 0.001)	5003 (–)	5347 (6.9; P < 0.001)	5622 (5.1; P < 0.01)	23,116 (–)	24,345 (5.3; P < 0.001)	9217 (–)	9708 (5.3; P < 0.001)	11,159 (15.0; P < 0.001)
All: 16–39*	371 (–)	362 (–2.4; P = 0.740)	142 (–)	152 (7.0; P = 0.561)	191 (25.7; P < 0.05)	1405 (–)	1627 (15.8; P < 0.001)	545 (–)	627 (15.1; P < 0.05)	790 (26.0; P < 0.001)
All: over 40*	10,778 (–)	12,430 (15.3; P < 0.001)	4861 (–)	5195 (6.9; P < 0.001)	5431 (4.5; P < 0.05)	21,711 (–)	22,718 (4.6; P < 0.001)	8672 (–)	9081 (4.7; P < 0.01)	10,369 (14.2; P < 0.001)
Female: overall	5492 (–)	6254 (13.9; P < 0.001)	2521 (–)	2629 (4.3; P = 0.132)	2756 (4.8; P = 0.084)	7877 (–)	8714 (10.6; P < 0.001)	3164 (–)	3473 (9.8; P < 0.001)	4118 (18.6; P < 0.001)
Female: 16–39	108 (–)	81 (–25.0; P < 0.05)	39 (–)	35 (–10.3; P = 0.648)	46 (31.4; P = 0.224)	304 (–)	408 (34.2; P < 0.001)	112 (–)	152 (35.7; P < 0.05)	214 (40.8; P < 0.01)
Female: over 40	5384 (–)	6173 (14.7; P < 0.001)	2482 (–)	2594 (4.5; P = 0.116)	2710 (4.5; P = 0.111)	7573 (–)	8306 (9.7; P < 0.001)	3052 (–)	3321 (8.8; P < 0.001)	3904 (17.6; P < 0.001)
Male: overall	5636 (–)	6537 (16.0; P < 0.001)	2473 (–)	2717 (9.9; P < 0.001)	2866 (5.5; P < 0.05)	15,137 (–)	15,630 (3.3; P < 0.01)	5993 (–)	6235 (4.0; P < 0.05)	7041 (12.9; P < 0.001)
Male: 16–39	260 (–)	280 (7.7; P = 0.390)	102 (–)	116 (13.7; P = 0.344)	145 (25.0; P = 0.073)	1095 (–)	1219 (11.3; P < 0.01)	430 (–)	475 (10.5; P = 0.135)	576 (21.3; P < 0.01)
Male: over 40	5376 (–)	6257 (16.4; P < 0.001)	2371 (–)	2601 (9.7; P < 0.01)	2721 (4.6; P = 0.100)	14,042 (–)	14,411 (2.6; P < 0.05)	5563 (–)	5760 (3.5; P = 0.064)	6465 (12.2; P < 0.001)

Corrected Table 1:

**Table:** 

Gender: Age Group	Cardiac Arrest, Counts (Percent change relative to previous year; P-value)					Acute Coronary Syndrome, Counts (Percent change relative to previous year; P-value)				
	Full year counts			January–May counts		Full year counts		January–May counts		
	2019	2020 (Percent change relative to 2019; P-value)	2019	2020 (Percent change relative to January–May 2019; P-value)	2021 (Percent change relative to January–May 2020; P-value)	2019	2020 (Percent change relative to 2019; P-value)	2019	2020 (Percent change relative to January–May 2019; P-value)	2021 (Percent change relative to January–May 2020; P-value)
All: Overall*	11,187 (–)	12,866 (15.0; P < 0.001)	4,994 (–)	5,379 (7.7; P < 0.001)	5,651 (5.1; P < 0.01)	23,313 (–)	24,501 (5.1; P < 0.001)	9,293 (–)	9,762 (5.1; P < 0.001)	11,204 (14.8; P < 0.001)
All: 16–39*	367 (–)	369 (0.5; P = 0.942)	136 (–)	152 (11.8; P = 0.347)	191 (25.7; P < 0.05)	1,418 (–)	1,642 (15.8; P < 0.001)	548 (–)	635 (15.9; P < 0.05)	791 (24.6; P < 0.001)
All: Over 40*	10,820 (–)	12,497 (15.5; P < 0.001)	4,858 (–)	5,227 (7.6; P < 0.001)	5,460 (4.5; P < 0.05)	21,895 (–)	22,859 (4.4; P < 0.001)	8,745 (–)	9,127 (4.4; P < 0.01)	10,413 (14.1; P < 0.001)
Female: Overall	5,496 (–)	6,282 (14.3; P < 0.001)	2,510 (–)	2,644 (5.3; P = 0.062)	2,759 (4.4; P = 0.118)	7,904 (–)	8,768 (10.9; P < 0.001)	3,167 (–)	3,483 (10.0; P < 0.001)	4,125 (18.4; P < 0.001)
Female: 16–39	104 (–)	87 (− 16.4; P = 0.220)	34 (–)	36 (5.9; P = 0.818)	45 (25.0; P = 0.321)	305 (–)	411 (34.8; P < 0.001)	112 (–)	154 (37.5; P < 0.01)	212 (37.7; P < 0.01)
Female: Over 40	5,392 (–)	6,195 (14.9; P < 0.001)	2,476 (–)	2,608 (5.3; P = 0.064)	2,714 (4.1; P = 0.146)	7,599 (–)	8,357 (10.0; P < 0.001)	3,055 (–)	3,329 (9.0; P < 0.001)	3,913 (17.5; P < 0.001)
Male: Overall	5,670 (–)	6,583 (16.1; P < 0.001)	2,475 (–)	2,734 (10.5; P < 0.001)	2,892 (5.8; P < 0.05)	15,307 (–)	15,732 (2.8; P < 0.05)	6,065 (–)	6,279 (3.5; P = 0.054)	7,079 (12.7; P < 0.001)
Male: 16–39	260 (–)	281 (8.1; P = 0.367)	101 (–)	115 (13.9; P = 0.342)	146 (27.0; P = 0.055)	1,107 (–)	1,231 (11.2; P < 0.05)	433 (–)	481 (11.1; P = 0.112)	579 (20.4; P < 0.01)
Male: Over 40	5,410 (–)	6,302 (16.5; P < 0.001)	2,374 (–)	2,619 (10.3; P < 0.001)	2,746 (4.9; P = 0.083)	14,200 (–)	14,501 (2.1; P = 0.076)	5,632 (–)	5,798 (3.0; P = 0.121)	6,500 (12.1; P < 0.001)

Incorrect Table 2:

**Table:** 

Variable	With stepwise BIC selection		Without feature selection	
	Adjusted incidence rate ratio (95% CI)	P-value	Adjusted incidence rate ratio (95% CI)	P-value
The bi-weekly cumulative counts of 1st and 2nd vaccine doses in the age group 16–39, normalized by the respective population size	3.33 (2.14–5.14)	< 0.001	2.12 (1.05–4.22)	< 0.05
The three-week cumulative new COVID-19 infection count among the age group 16–39, normalized by the respective population size	–	–	27.37 (0.05–13,177.26)	0.295
Call type: Acute coronary syndrome	1 [Reference]	–	1 [Reference]	–
Call type: Cardiac arrest	0.24 (0.22–0.26)	< 0.001	0.24 (0.22–0.26)	< 0.001
Week not during a COVID-19 public health advisory	1 [Reference]	–	1 [Reference]	–
Week during a COVID-19 public health advisory	–	–	0.94 (0.85–1.04)	0.233
Year: 2019	0.89 (0.83–0.94)	< 0.001	0.82 (0.74–0.91)	< 0.001
Year: 2020	–	–	0.92 (0.83–1.03)	0.146
Year: 2021	1 [Reference]	–	1 [Reference]	–

Corrected Table 2:

**Table:** 

Variable	With Stepwise BIC selection		Without Feature Selection	
	Adjusted incidence rate ratio (95% CI)	P-value	Adjusted incidence rate ratio (95% CI)	P-value
The bi-weekly cumulative counts of 1st and 2nd vaccine doses in the age group 16–39, normalized by the respective population size	3.32 (2.12–5.14)	< 0.001	2.20 (1.06–4.56)	< 0.05
The three-week cumulative new COVID-19 infection count among the age group 16–39, normalized by the respective population size	–	–	26.94 (0.03–20,067.17)	0.330
Call type: Acute Coronary Syndrome	1 [Reference]	–	1 [Reference]	–
Call type: Cardiac arrest	0.24 (0.22–0.25)	< 0.001	0.24 (0.22–0.25)	< 0.001
Week not during a COVID-19 public health advisory	1 [Reference]	–	1 [Reference]	–
Week during a COVID-19 public health advisory	–	–	0.94 (0.85–1.04)	0.224
Year: 2019	0.88 (0.83–0.94)	< 0.001	0.83 (0.75–0.92)	< 0.001
Year: 2020	–	–	0.94 (0.84–1.05)	0.245
Year: 2021	1 [Reference]	–	1 [Reference]	–

Incorrect Table 3:

**Table:** 

Variable	With stepwise BIC selection		Without feature selection	
	Adjusted incidence rate ratio (95% CI)	P-value	Adjusted incidence rate ratio (95% CI)	P-value
The bi-weekly cumulative counts of 1st and 2nd vaccine doses per age group, normalized by the respective population size	1.79 (1.43–2.25)	< 0.001	1.92 (1.34–2.76)	< 0.001
The three-week cumulative new COVID-19 infection count per age group, normalized by the respective population size	–	–	6.21 (0.001–24,098.97)	0.668
Age group: Below 40	1 [Reference]	–	1 [Reference]	–
Age group: 40 and above	30.95 (28.89–33.21)	< 0.001	31.05 (28.90–33.41)	< 0.001
Week not during a COVID-19 public health advisory	1 [Reference]	–	1 [Reference]	–
Week during a COVID-19 public health advisory	–	–	0.98 (0.92–1.05)	0.639
Year: 2019	0.90 (0.86–0.94)	< 0.001	0.93 (0.87–0.99)	< 0.05
Year: 2020	–	–	1.04 (0.97–1.12)	0.233
Year: 2021	1 [Reference]	–	1 [Reference]	–

Corrected Table 3:

**Table:** 

Variable	With Stepwise BIC selection		Without Feature Selection	
	Adjusted incidence rate ratio (95% CI)	P-value	Adjusted incidence rate ratio (95% CI)	P-value
The bi-weekly cumulative counts of 1st and 2nd vaccine doses per age group, normalized by the respective population size	1.79 (1.43–2.23)	< 0.001	1.94 (1.36–2.76)	< 0.001
The three-week cumulative new COVID-19 infection count per age group, normalized by the respective population size	–	–	5.49 (0.001–19,001.98)	0.685
Age Group: Below 40	1 [Reference]	–	1 [Reference]	–
Age Group: 40 and above	31.08 (29.01–33.33)	< 0.001	31.16 (29.01–33.52)	< 0.001
Week not during a COVID-19 public health advisory	1 [Reference]	–	1 [Reference]	–
Week during a COVID-19 public health advisory	–	–	0.98 (0.92–1.05)	0.630
Year: 2019	0.89 (0.86–0.93)	< 0.001	0.92 (0.86–0.99)	< 0.05
Year: 2020	–	–	1.05 (0.98–1.12)	0.178
Year: 2021	1 [Reference]	–	1 [Reference]	–

Additionally, in the Supplementary Information, subheading ‘2.1. EMS calls and study population’,

“During the study period (January 1st, 2019, to June 20th, 2021), a total of 33,377 and 63,224 CA and ACS calls occurred, respectively. Of which 2,730 (8.2% of 33,377) and 2,785 (4.4% of 63,224) CA and ACS calls, respectively, had a missing age value and were excluded from the analysis. Among the calls with no missing age values, 385 and 41 CA and ACS calls were below the age of 16, respectively, and were also excluded from the analysis, resulting in the final 30,262 CA calls and 60,398 ACS calls included in the study population.”

now reads:

“During the study period (January 1st, 2019, to June 20th, 2021), a total of 32,884 and 63,322 CA and ACS calls occurred, respectively. Of which 2,005 (6.5% of 32,884) and 2,281 (3.6% of 63,322) CA and ACS calls, respectively, had a missing age value and were excluded from the analysis. Among the calls with no missing age values, 398 and 40 CA and ACS calls were below the age of 16, respectively, and were also excluded from the analysis, resulting in the final 30,481 CA calls and 61,001 ACS calls included in the study population.”

In the Supplementary Information, subheading ‘2.2. Year-to-year trends in cardiac arrest patients that died on scene and received resuscitation’,

“Among the 16–39 age group, the percent of patients that died on scene increased significantly from 2019 to 2020 for the full year (52.8% to 60.5%; P<0.001). The death rate remained elevated during January–May 2021 at 61.3% and was not significantly different (P=0.460) from the 65.1% death rate during the same time in 2020. The proportion of patient from ages 16–39 that received resuscitation also increased in significantly from 2019 to 2020 (41.5% to 54.4%; P<0.001), and remained elevated in January–May 2021 (53.9%).”

now reads:

“Among the 16–39 age group, the percent of patients that died on scene increased significantly from 2019 to 2020 for the full year (50.7–59.9%; P < 0.05). The death rate remained elevated during January–May 2021 at 58.1% and was not significantly different (P=0.185) from the 65.1% death rate during the same time in 2020. The proportion of patients from ages 16–39 that received resuscitation also increased in significantly from 2019 to 2020 (43.9–55.8%; P < 0.01), and remained elevated in January–May 2021 (56.5%).”

In the Supplementary Information, subheading ‘2.3 Correlation of new COVID-19 infections counts with cardiac arrest and acute coronary syndrome calls’,

“In the explored scenarios, the cumulative new COVID-19 case counts were not significantly correlated with CA call counts or the sum of CA and ACS call counts.”

now reads:

“In the explored scenarios, the cumulative new COVID-19 case counts were not significantly correlated with CA call counts and were significantly correlated with the sum of CA and ACS call counts.”

Additionally, to improve clarity of exposition, Figures 1 and 2 in the body of the paper, and Figures 1–4 in the supplementary appendix are presented with a modified y-axis scale, starting from 0. The original Figures 1 and 2 are shown below and the original Supplementary Information file is appended to this notice, for reference.

**Figure 1:**
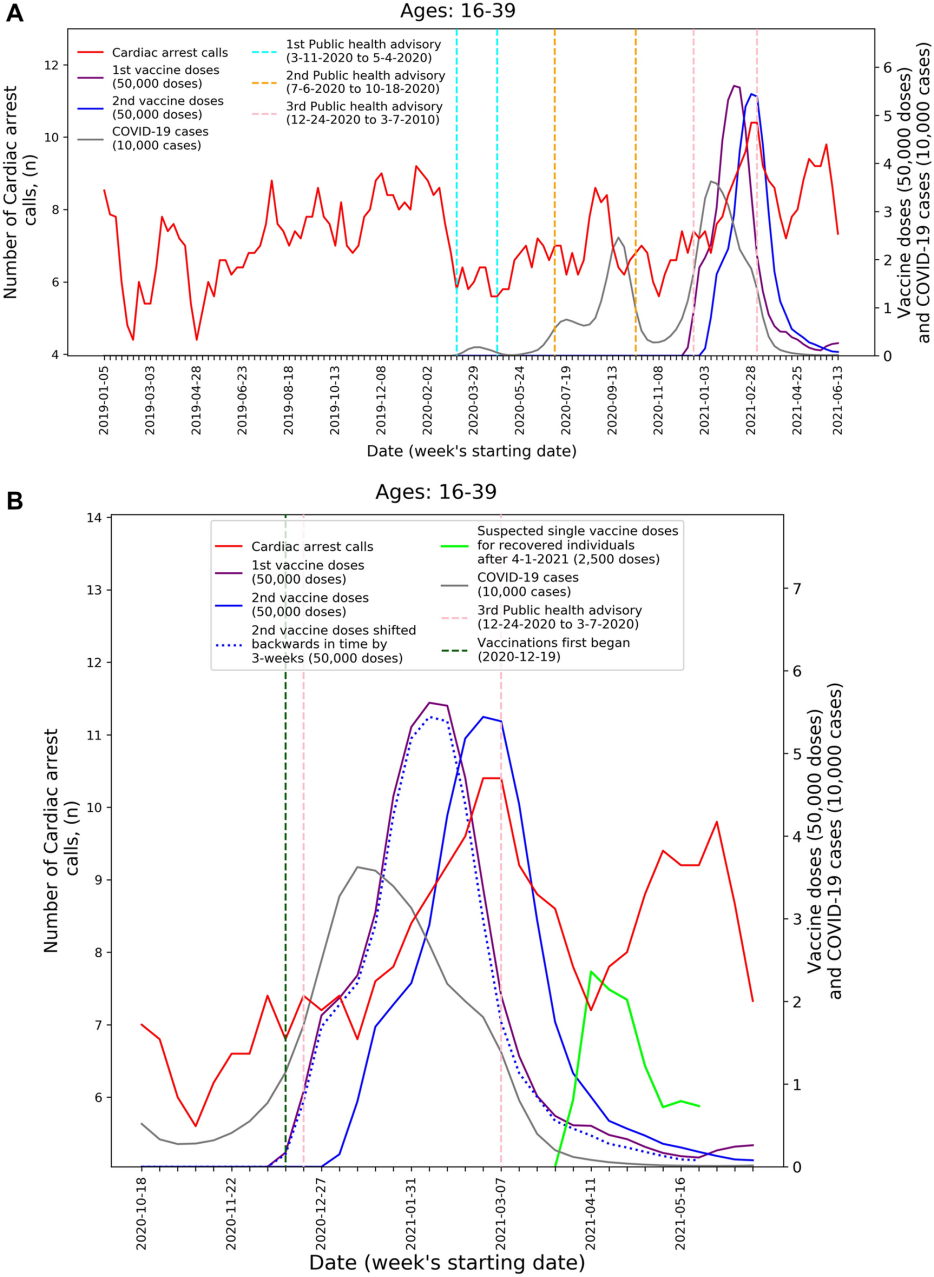
Weekly counts of cardiac arrest calls (five-week centered moving-average), COVID-19 cases (three-week centered moving-average), and vaccination doses (three-week centered moving-average) for those between 16 and 39 during: (**A**) the study period (January 1st, 2019, to June 20th, 2021) and (**B**) the third COVID-19 wave and vaccination distribution period (October 18th, 2020, to June 20th, 2021). *COVID-19* Coronavirus disease 2019.

**Figure 2:**
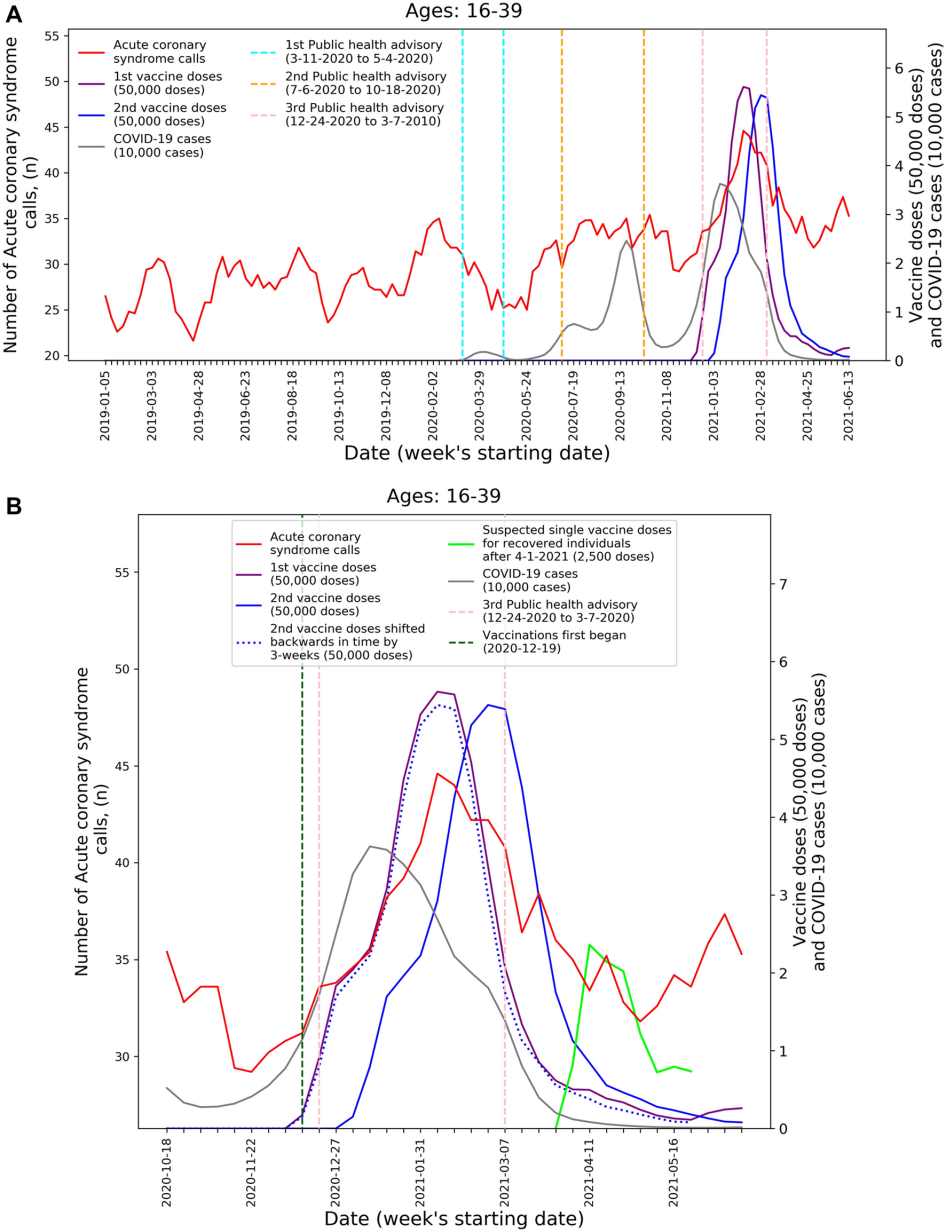
Weekly counts of acute coronary syndrome calls (five-week centered moving-average), COVID-19 cases (three-week centered moving-average), and vaccination doses (three-week centered moving-average) for those between 16 and 39 during: (**A**) the study period (January 1st, 2019, to June 20th, 2021) and (**B**) the third COVID-19 wave and vaccination distribution period (October 18th, 2020, to June 20th, 2021). *COVID-19* Coronavirus disease 2019.

Finally, documentation of all the data files, templates, codes and respective output files used in the analyses are now deposited on Figshare. Data Availability section of the paper was updated to reflect this.

“The COVID-19 and vaccination rate datasets generated and analysed during the current study are available at https://data.gov.il/dataset/covid-19. EMS call count data are not publicly available as they are derived from national clinical records. Due to national and organizational data privacy regulations this data cannot be shared openly.”

now reads:

“The COVID-19 and vaccination rate datasets generated and analysed during the current study are available at https://data.gov.il/dataset/covid-19. EMS call count data are not publicly available as they are derived from national clinical records. Due to national and organizational data privacy regulations this data cannot be shared openly. All the data files, templates, codes and respective output files used in the analyses are deposited on Figshare and can be accessed at https://doi.org/10.6084/m9.figshare.23805954.v1.”

The original version of this Article and the Supplementary Information file have been corrected.

### Supplementary Information

Supplementary Information.

