# Author Correction: Increased emergency cardiovascular events among under-40 population in Israel during vaccine rollout and third COVID-19 wave

**DOI:** 10.1038/s41598-026-43277-2

**Published:** 2026-03-12

**Authors:** Christopher L. F. Sun, Eli Jaffe, Retsef Levi

**Affiliations:** 1 Sloan School of Management, Massachusetts Institute of Technology, 100 Main Street, Cambridge, MA 02142-1347 USA; 2 Healthcare Systems Engineering, Massachusetts General Hospital, Boston, MA USA; 3 Israel National Emergency Medical Services (Magen David Adom), Tel Aviv-Jaffo, Israel; 4 Ben Gurion University of the Negev, Beer Sheva, Israel

Correction to: *Scientific Reports* 10.1038/s41598-022-10928-z, published online 28 April 2022

The original version of this Article contained a typographical error in the Results section in which the percentage of patients aged 16–39 receiving their second vaccination dose was reported as 32.2% rather than 42.2%.

This has now been updated and the Results section,

“Among the 5,506,398 patients receiving their 1st vaccination dose and 5,152,417 patients receiving their 2nd vaccination dose, 2,382,864 (43.3%) and 2,176,172 (32.2%) patients were of age 16–39, respectively.”

now reads:

“Among the 5,506,398 patients receiving their 1st vaccination dose and 5,152,417 patients receiving their 2nd vaccination dose, 2,382,864 (43.3%) and 2,176,172 (42.2%) patients were of age 16–39, respectively.”

The original Article has been corrected.

