# Supplementary material for: Increased emergency cardiovascular events among under-40 population in Israel during vaccine rollout and third COVID-19 wave
Source: Sci Rep. 2022 Apr 28;12:6978. doi: 10.1038/s41598-022-10928-z (PMC9048615; doi:10.1038/s41598-022-10928-z)
Supplement: Supplementary file 1 — Supplementary Information. [file 41598_2022_10928_MOESM1_ESM.docx]

**Supplemental Material**

Contents

[**1.0 Supplemental Methods** 2](#_Toc81211895)

[1.1 Israel National Emergency Medical Services dataset details 2](#_Toc81211896)

[1.2 Year-to-year trends in cardiac arrest characteristics 2](#_Toc81211897)

[1.3 Negative binomial regression model equations 2](#_Toc81211898)

[**2.0 Supplemental Results** 3](#_Toc81211899)

[2.1. EMS calls and study population 3](#_Toc81211900)

[2.2. Year-to-year trends in cardiac arrest patients that died on scene and received resuscitation 3](#_Toc81211901)

[2.3. Correlation of new COVID-19 infections counts with cardiac arrest and acute coronary syndrome calls 6](#_Toc81211902)

[2.4 Visualizations of cardiac arrest and acute coronary syndrome calls for all ages and those 40 and above 6](#_Toc81211903)

[2.5. Negative binomial regression model sensitivity analysis: Varying the count period of the new COVID-19 infections count time series covariate 11](#_Toc81211904)

# **1.0 Supplemental Methods**

## 1.1 Israel National Emergency Medical Services dataset details

*Israel National Emergency Medical Services call data fields*

The Israel National Emergency Medical Services (IMES) dataset contains information on the date and time of the call, the suspected call type code at dispatch, retrospectively confirmed call type by the EMS team, age, gender, cardiac rhythm, consciousness status, if resuscitation was required (e.g., defibrillation or cardiopulmonary resuscitation delivered), call disposition (e.g., transferred to a hospital, refused transport, declared dead on scene) of each received emergency call.

*Cardiac arrest and acute coronary syndrome call type codes and data preprocessing*

The retrospectively verified call type codes were utilized to categorize cardiac arrest (CA) and acute coronary syndrome (ACS) calls analyzed in the study. The cardiac arrests calls analyzed in the study included calls coded as *Cardiac Arrest* as well as calls that required resuscitation (as defined above) with a call code type of *Cardiogenic shock*, *Changes in ECG*, *Determining death/declaring death*, *Other arrhythmias, Ventricular* *tachycardia (PSVT),* and *Ventricular tachycardia (VT)*. The ACS calls included calls coded as *Acute coronary syndrome* and *Myocardial infarction*.

## 1.2 Year-to-year trends in cardiac arrest characteristics

For CA events in each age group (16-39, over-40 or all-ages), the percent of patients that died on scene as well as received resuscitation were calculated respect to the full calendar year (2019-2020) and the months January-May (2019-2021). The statistical significance of the year-to-year changes in the portions of each these two characteristics were calculated using a chi-squared test.

## 1.3 Negative binomial regression model equations

*Regression Model 1*

The following formula describes the first negative binomial regression model, *Model 1*, which regresses the respective time-series of CA weekly count and the ACS weekly count in age group 16-39 as the dependent variable.

log(Weekly EMS call counts in the age group 16-39_Call type_) = β_0_ + β_1_(Two-week cumulative counts of 1st and 2nd vaccine doses administered in the age group 16-39 normalized by the 16-39 population size) + β_2_(Three-week cumulative new COVID-19 infection counts in the age group 16-39 normalized by the 16-39 population size) + β_3_(Call type of Cardiac Arrest _Call type_) + β_4_(Week is included in a period of national public health lockdown) + β_5_(Week is during 2019) + β_6_(Week is during 2020)

*Regression Model 2*

The following formula describes the second negative binomial regression model, *Model 2*, which regresses the respective time-series of CA weekly counts of age groups 16-39 and over 40, as the dependent variable.

log(Weekly cardiac arrest call counts_Age group_) = β_0_ + β_1_(Two-week cumulative counts of 1st and 2nd vaccine doses administered in the respective age group normalized by the respective population size_Age group_) + β_2_(Three-week cumulative new COVID-19 infection counts in the respective age group normalized by the age group’s population size_Age group_) + β_3_(Age group of 40 and above_Age group_) + β_4_(Week is included in a period of national public health lockdown) + β_5_(Week is during 2019) + β_6_(Week is during 2020)

# **2.0 Supplemental Results**

## 2.1. EMS calls and study population

During the study period (January 1st, 2019, to June 20th, 2021), a total of 32,884 and 63,322 CA and ACS calls occurred, respectively. Of which 2,005 (6.5% of 32,884) and 2,281 (3.6% of 63,322) CA and ACS calls, respectively, had a missing age value and were excluded from the analysis. Among the calls with no missing age values, 398 and 40 CA and ACS calls were below the age of 16, respectively, and were also excluded from the analysis, resulting in the final 30,481 CA calls and 61,001 ACS calls included in the study population.

## 2.2. Year-to-year trends in cardiac arrest patients that died on scene and received resuscitation

Supplemental Tables 1 and 2 summarizes the year-to-year changes in CA patients that died on scene and received resuscitation, respectively. Among the 16-39 age group, the percent of patients that died on scene increased significantly from 2019 to 2020 for the full year (50.7–59.9%; P < 0.05). The death rate remained elevated during January-May 2021 at 58.1% and was not significantly different (P=0.185) from the 65.1% death rate during the same time in 2020. The proportion of patient from ages 16-39 that received resuscitation also increased in significantly from 2019 to 2020 (43.9–55.8%; P < 0.01), and remained elevated in January-May 2021 (56.5%).

| Gender: Age Group | Cardiac arrest: Percent died on scene,  (Death Count/Total Count; P-value of change in proportion relative to previous year) | | | | |
| --- | --- | --- | --- | --- | --- |
|  | **Full year** | | **January – May** | | |
|  | **2019**  **(Death Count/Total Count; - )** | **2020**  **(Death Count/Total Count; P-value of change relative to 2019)** | **2019**  **(Death Count/Total Count; - )** | **2020**  **(Death Count/Total Count; P-value of change relative to January-May 2019)** | **2021**  **(Death Count/Total Count; P-value of change relative to January-May 2020)** |
| All: Overall^*^ | 74.2  (8,298/11,187; -) | 77.5  (9,967/12,866; P<0.001) | 73.3  (3,662/4,994; -) | 78.1  (4,200/5,379; P<0.001) | 72.9  (4,119/5,651; P<0.001) |
| All: 16-39^*^ | 50.7  (186/367; -) | 59.9  (221/369; P<0.05) | 48.5  (66/136; -) | 65.1  (99/152; P<0.01) | 58.1  (111/191; P=0.185) |
| All: Over 40^*^ | 75.0  (8,112/10,820; -) | 78.0  (9,746/12,497; P<0.001) | 74.0  (3,596/4,858; -) | 78.5  (4,101/5,227; P<0.001) | 73.4  (4,008/5,460; P<0.001) |
| Female: Overall | 78.2  (4,295/5,496; -) | 80.8  (5,075/6,282; P<0.001) | 76.7  (1,924/2,510; -) | 81.7  (2,161/2,644; P<0.001) | 76.4  (2,108/2,759; P<0.001) |
| Female: 16-39 | 57.7 (60/104; -) | 57.5 (50/87; P=0.975) | 38.2 (13/34; -) | 66.7 (24/36; P<0.05) | 53.3  (24/45; P=0.225) |
| Female: Over 40 | 78.5  (4,235/5,392; -) | 81.1  (5,025/6,195; P<0.001) | 77.2  (1,911/2,476; -) | 81.9  (2,137/2,608; P<0.001) | 76.8  (2,084/2,714; P<0.001) |
| Male: Overall | 70.6  4,000/5,670; -) | 74.3  (4,892/6,583; P<0.001) | 70.1  (1,735/2,475; -) | 74.6  (2,039/2,734; P<0.001) | 69.5  (2,011/2,892; P<0.001) |
| Male: 16-39 | 48.1  (125/260; -) | 60.9  (171/281; P<0.01) | 51.5  (52/101; -) | 65.2  (75/115; P<0.05) | 59.6  (87/146; P=0.352) |
| Male: Over 40 | 71.6  (3,875/5,410; -) | 74.9  (4,721/6,302; P<0.001) | 70.9  (1,683/2,374; -) | 75.0  (1,964/2,619; P<0.01) | 70.1  (1,924/2,746; P<0.001) |

**Supplemental Table 1. Year-to-year trends in the percent of cardiac arrest calls where the patient died on scene by age group and gender. Each cell shows the percent of calls where the patient died on scene during the respective time period, age group, and gender with the P-value corresponding to the change in proportion from the previous year in the parenthesis (e.g., significance of change from 2020 to 2021). The changes in proportions were** **calculated across the same duration per year (i.e., either across the full year or across the January-May period). For counts during 2019, no P-value is reported.**

**^*^Counts in the All category includes calls with missing gender variable values. Number of calls with missing gender values: Cardiac arrest: N = 22.**

| Gender: Age Group | Cardiac arrest: Percent received resuscitation,  (Resuscitation Count/Total Count; P-value of change of proportion relative to previous year) | | | | |
| --- | --- | --- | --- | --- | --- |
|  | **Full year** | | **January – May** | | |
|  | **2019 (Resuscitation Count/Total Count; - )** | **2020 (Resuscitation Count/Total Count; P-value of change relative to 2019)** | **2019**  **(Resuscitation Count/Total**  **Count; - )** | **2020**  **(Resuscitation Count/Total Count; P-value of change relative to January-May 2019)** | **2021**  **(Resuscitation Count/Total Count; P-value of change relative to January-May 2020)** |
| All: Overall^*^ | 28.2  (3,158/11,187; -) | 35.0  (4,506/12,866; P<0.001) | 25.3  (1,264/4,994; -) | 36.1  (1,943/5,379; P<0.001) | 37.2  (2,100/5,651; P=0.257) |
| All: 16-39^*^ | 43.9  (161/367; -) | 55.8  (206/369; P<0.01) | 43.4  (59/136; -) | 55.9  (85/152; P<0.05) | 56.5  (108/191; P=0.908) |
| All: Over 40^*^ | 27.7  (2,997/10,820; -) | 34.4  (4,300/12,497; P<0.001) | 24.8  (1,205/4,858; -) | 35.6  (1,858/5,227; P<0.001) | 36.5  (1,992/5,460; P=0.313) |
| Female: Overall | 23.9  (1,314/5,496; -) | 30.4  (1,907/6,282; P<0.001) | 21.3  (535/2,510; -) | 30.4  (803/2,644; P<0.001) | 31.6  (872/2,759; P=0.327) |
| Female: 16-39 | 46.2  (48/104; -) | 50.6  (44/87; P=0.543) | 35.3  (12/34; -) | 50.0  (18/36; P=0.214) | 62.2  (28/45; P=0.270) |
| Female: Over 40 | 23.5  (1,266/5,392; -) | 30.1  (1,863/6,195; P<0.001) | 21.1  (523/2,476; -) | 30.1  (785/2,608; P<0.001) | 31.1  (844/2,714; P=0.430) |
| Male: Overall | 32.5  (1,842/5,670; -) | 39.5  (2,598/6,583; P<0.001) | 29.4  (728/2,475; -) | 41.7  (1,139/2,734; P<0.001) | 42.5  (1,228/2,892; P=0.543) |
| Male: 16-39 | 43.1  (112/260; -) | 57.3  (161/281; P<0.001) | 46.5  (47/101; -) | 57.4  (66/115; P=0.111) | 54.8  (80/146; P=0.675) |
| Male: Over 40 | 32.0  (1,730/5,410; -) | 38.7  (2,437/6,302; P<0.001) | 28.7  (681/2,374; -) | 41.0  (1,073/2,619; P<0.001) | 41.8  (1,148/2,746; P=0.534) |

**Supplemental Table 2. Year-to-year trends in the percent of cardiac arrest calls where the patient received resuscitation by age group and gender. Each cell shows the percent of calls where the patient received resuscitation during the respective time period, age group, and gender with the P-value corresponding to the change in proportion from the previous year in the parenthesis (e.g., significance of change from 2020 to 2021). The changes in proportions were calculated across the same duration per year (i.e., either across the full year or across the January-May period). For counts during 2019, no P-value is reported.**

**^*^Counts in the All category includes calls with missing gender variable values. Number of calls with missing gender values: Cardiac arrest: N = 22.**

## 2.3. Correlation of new COVID-19 infections counts with cardiac arrest and acute coronary syndrome calls

Supplemental Table 3 shows the Spearman rank correlation between the weekly cumulative new COVID-19 infections counts, with the cumulative count period varied from one to six weeks, and both the weekly CA call counts and the sum of weekly CA and ACS calls counts, all within the 16-39 age group. In the explored scenarios, the cumulative new COVID-19 case counts were not significantly correlated with CA call counts and were significantly correlated with the sum of CA and ACS call counts.

| Count period for the cumulative new COVID-19 infections count in the age group 16-39 | Weekly count of cardiac arrest calls in the age group 16-39 | | Weekly cumulative count of cardiac arrest and acute coronary syndrome calls in the age group 16-39 | |
| --- | --- | --- | --- | --- |
|  | **Spearman rank correlation coefficient** | **P-value** | **Spearman rank correlation coefficient** | **P-value** |
| One week (current week) | 0.066 | 0.458 | 0.396 | <0.001 |
| Two weeks | 0.068 | 0.443 | 0.403 | <0.001 |
| Three weeks | 0.076 | 0.392 | 0.410 | <0.001 |
| Four weeks | 0.074 | 0.408 | 0.415 | <0.001 |
| Five weeks | 0.074 | 0.406 | 0.417 | <0.001 |
| Six weeks | 0.074 | 0.407 | 0.418 | <0.001 |

**Supplemental Table 3. Spearman rank correlation between the new COVID-19 infections counts and cardiac arrest and acute coronary syndrome calls.**

## 2.4 Visualizations of cardiac arrest and acute coronary syndrome calls for all ages and those 40 and above

Supplemental Figures 1-4 show the changes in the CA and ACS call counts for all ages and the 40 and above age group over the study period. Both CA and ACS calls start increasing in late December 2021 during the period of mass vaccine distribution and the third COVID-19 infection wave. Notably, the CA calls seem to track the 2^nd^ dose counts (blue line) closely, with both peaking in January 2021 and experiencing a halt in the decrease of counts in February 2021.

| **A** | 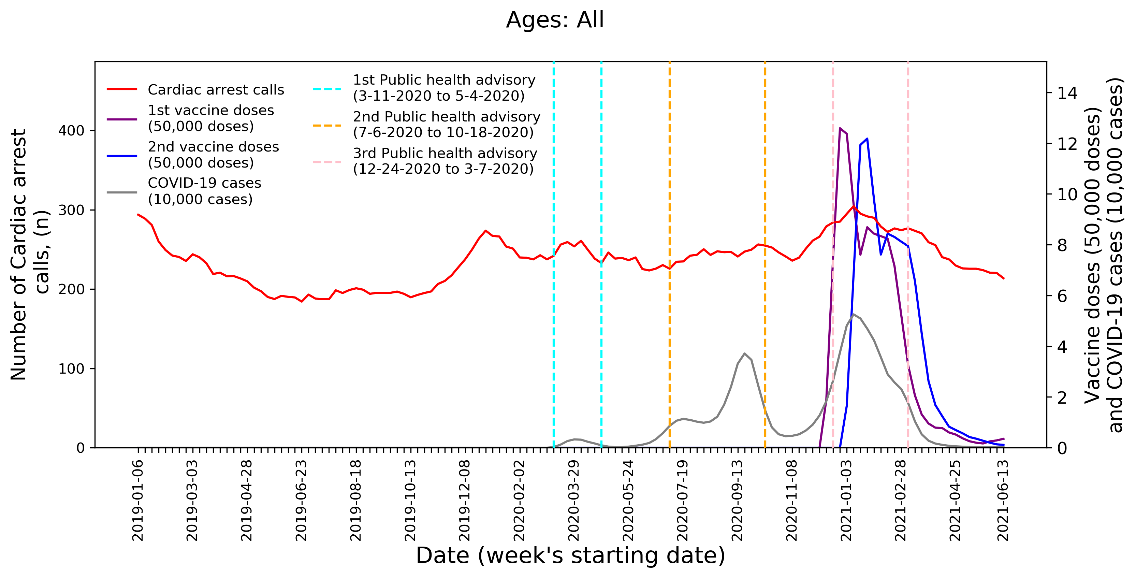 |
| --- | --- |
| **B** | 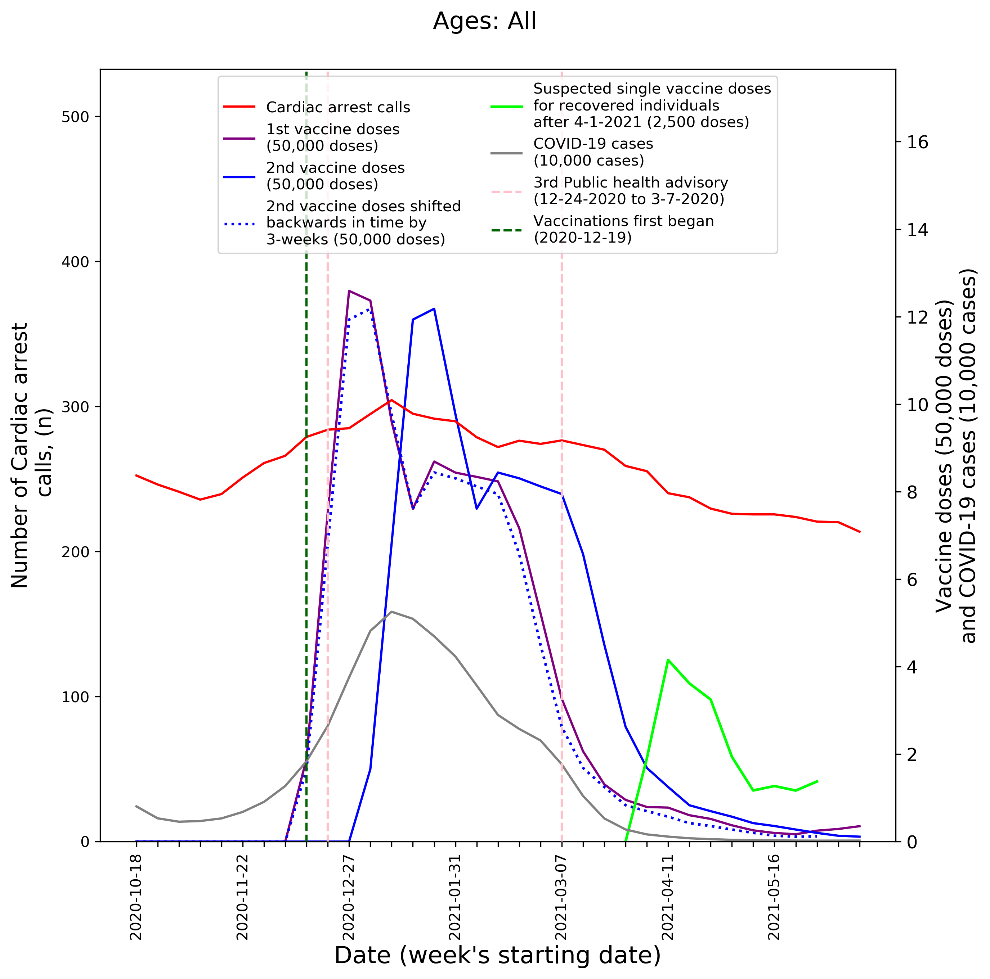 |

**Supplemental Figure 1. Weekly count of cardiac arrest calls (five-week centered moving-average), COVID-19 cases (three-week centered moving-average), and vaccination doses (three-week centered moving-average) over all ages during A) the study period (January 1^st^ 2019 to June 20^th^ 2021) and B) the third COVID-19 wave and vaccination distribution period (October 18^th^ 2020 to June 20^th^ 2021).**

| **A** | 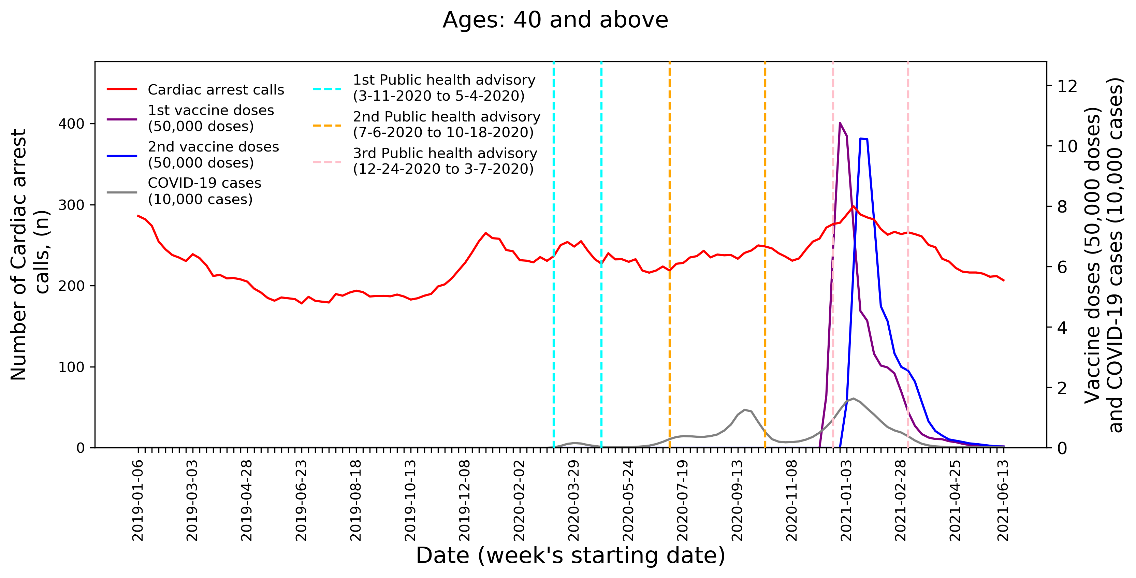 |
| --- | --- |
| **B** | 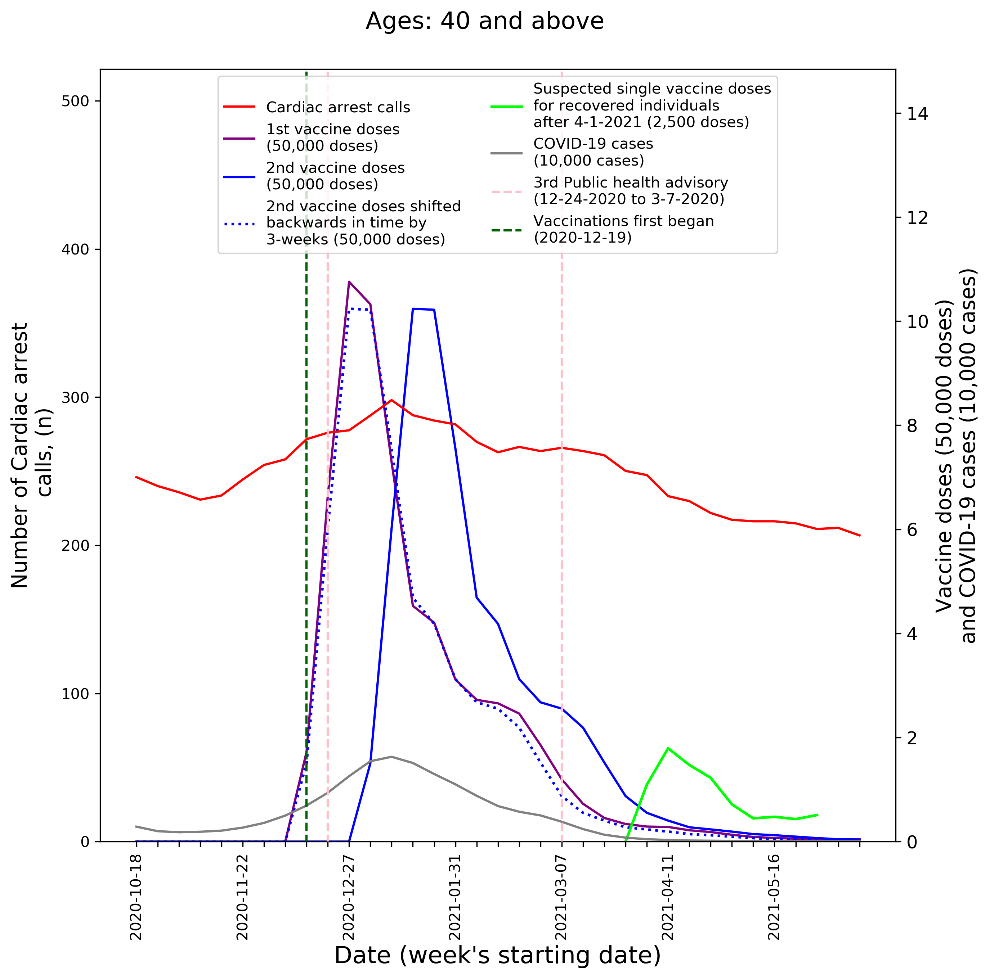 |

**Supplemental Figure 2. Weekly count of cardiac arrest calls (five-week centered moving-average), COVID-19 cases (three-week centered moving-average), and vaccination doses (three-week centered moving-average) of those 40 and above during A) the study period (January 1^st^ 2019 to June 20^th^ 2021) and B) the third COVID-19 wave and vaccination distribution period (October 18^th^ 2020 to June 20^th^ 2021).**

| **A** | 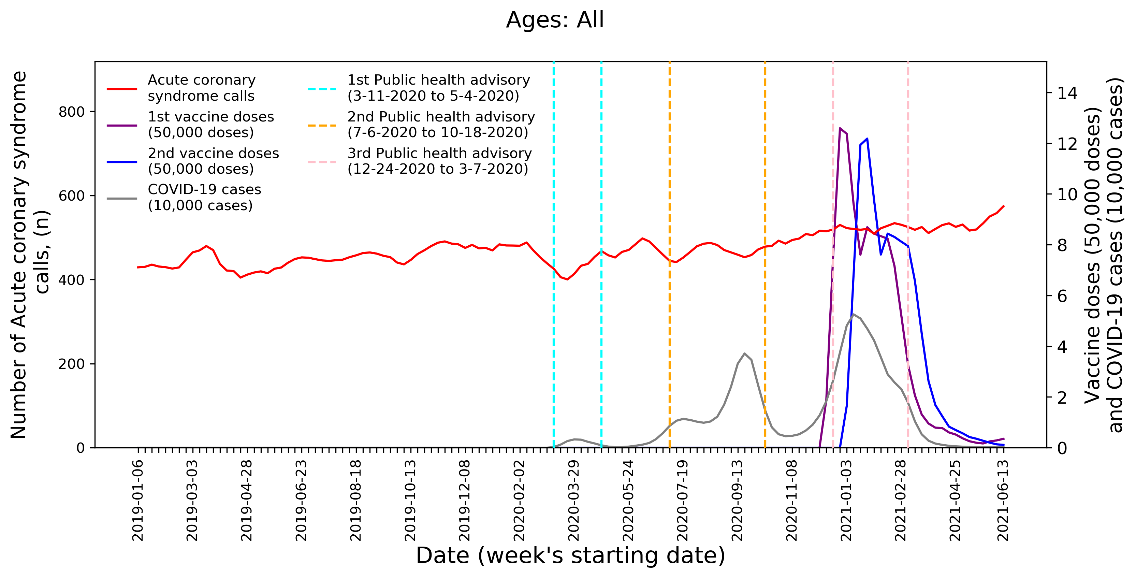 |
| --- | --- |
| **B** | 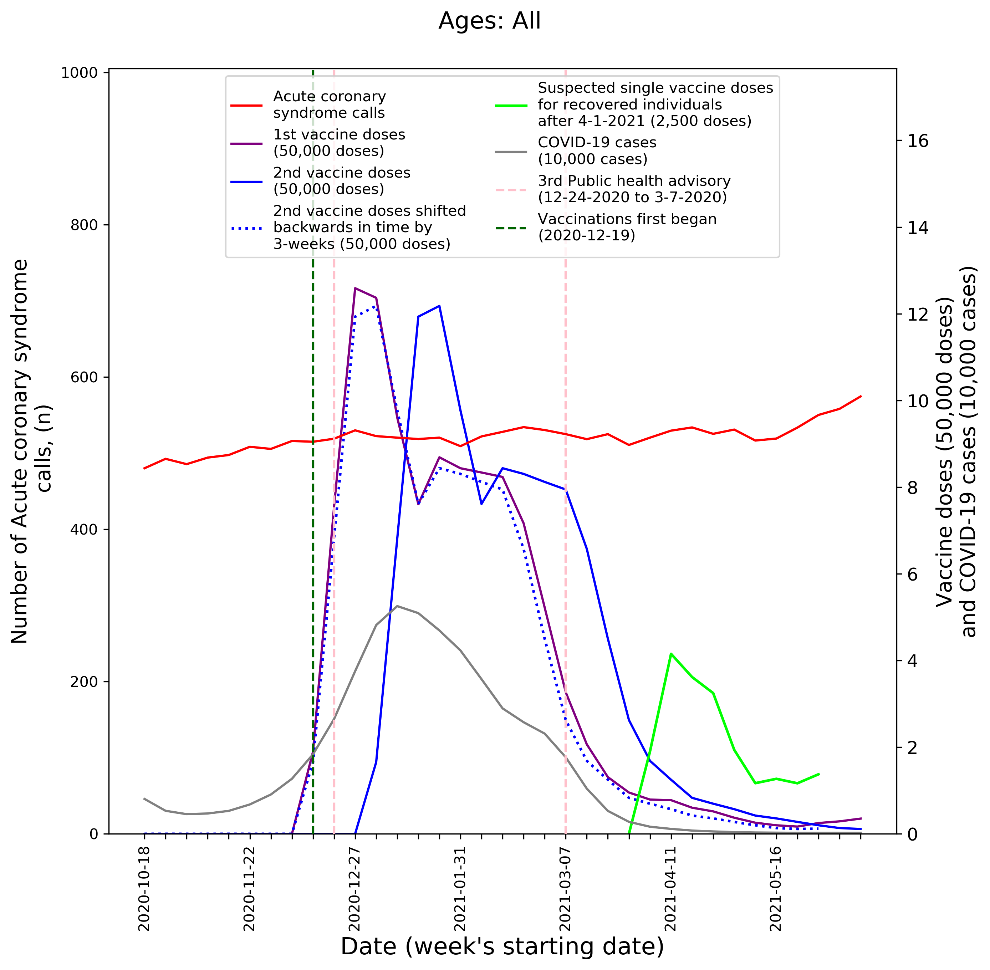 |

**Supplemental Figure 3. Weekly count of acute coronary syndrome calls (five-week centered moving-average), COVID-19 cases (three-week centered moving-average), and vaccination doses (three-week centered moving-average) over all ages during A) the study period (January 1^st^ 2019 to June 20^th^ 2021) and B) the third COVID-19 wave and vaccination distribution period (October 18^th^ 2020 to June 20^th^ 2021).**

| **A** | 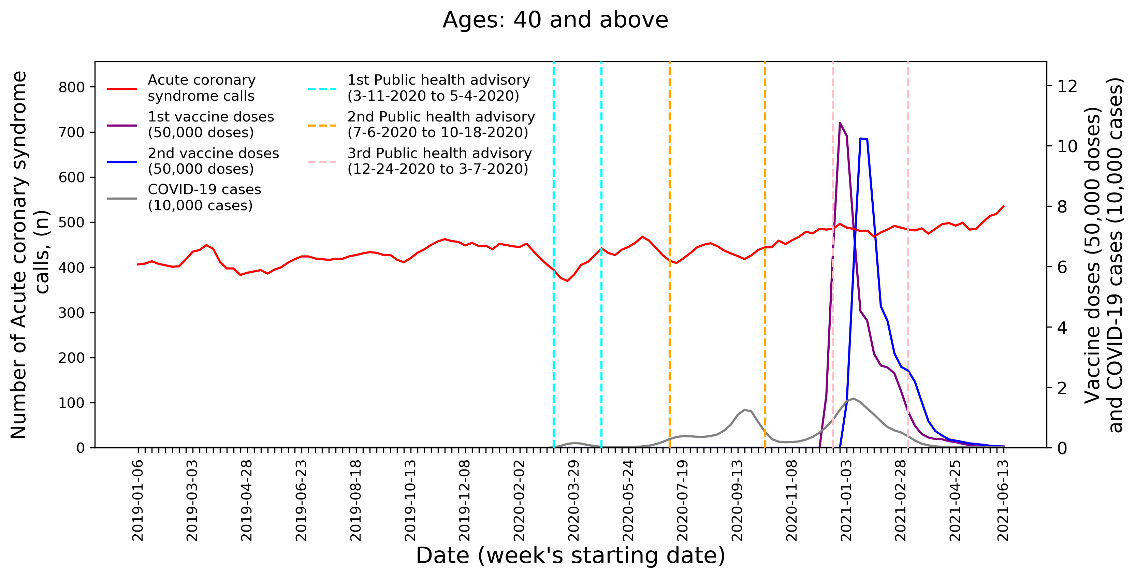 |
| --- | --- |
| **B** | 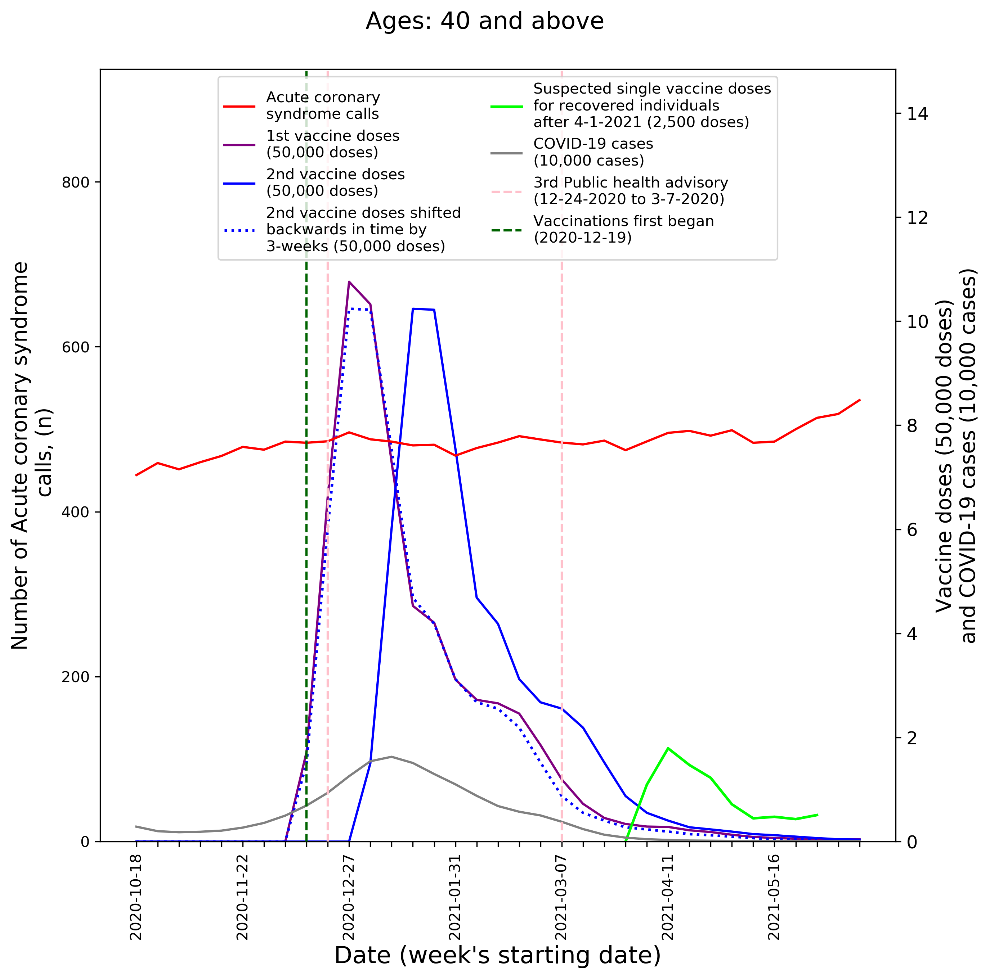 |

**Supplemental Figure 4. Weekly count of acute coronary syndrome calls (five-week centered moving-average), COVID-19 cases (three-week centered moving-average), and vaccination doses (three-week centered moving-average) of those 40 and above during A) the study period (January 1^st^ 2019 to June 20^th^ 2021) and B) the third COVID-19 wave and vaccination distribution period (October 18^th^ 2020 to June 20^th^ 2021).**

## 2.5. Negative binomial regression model sensitivity analysis: Varying the count period of the new COVID-19 infections count time series covariate

Supplemental Tables 4-7 summarize the results of the two negative binomial regression models (*Model 1* and *Model 2* as described in the methods section) with and without stepwise Bayesian information criterion (BIC) feature selection, while varying the count period of the new COVID-19 infection count time-series covariate from one to six weeks. Specifically, the tables show the adjusted beta coefficients and p-values of the cumulative new COVID-19 case count time-series covariate (with varied count periods from one to six weeks) and the bi-weekly cumulative count of 1^st^ and 2^nd^ vaccine dose time-series covariate for each model’s respective dependent variables. Each row in the summary tables represents a distinct regression model (i.e., each table shows the results of 6 different models, each using the corresponding COVID-19 case count and vaccine dose count covariates indicated in the first two columns of the tables, along with all other covariates described in the methods section of the main paper).

For both *Model 1* and *Model 2* using BIC feature selection, regardless of the count period of the new COVID-19 time series counts, the COVID-19 case count covariate was never selected as a statistically significant predictor of each model’s respective dependent variable (Supplemental Tables 4 and 6). For reference, the time-series of the cumulative count of 1^st^ and 2^nd^ vaccine doses was selected as a statistically significant predictor in each model and had a significant positive relationship with their respective dependent variables. Furthermore, without feature selection, the time-series new COVID-19 counts were never significantly associated with each model’s dependent variable, regardless of the covariate’s count period (Supplemental Tables 5 and 7). As expected, the BIC model performance metrics were more favorable (i.e., lower in value) among models that employed feature selection compared to the respective equivalent models that did not use feature selection.

| Count period for the cumulative count of new COVID-19 cases covariate | Count period for the cumulative count of 1^st^ and 2^nd^ vaccine doses covariate | Model BIC | Model Adjusted R^2^ | New COVID-19 case count covariate | | Cumulative count of vaccine doses covariate | |
| --- | --- | --- | --- | --- | --- | --- | --- |
|  |  |  |  | **Model beta coefficient** | **P-value** | **Model beta coefficient** | **P-value** |
| One week (current week) | **Two weeks** | 1450.9 | 0.872 | Not selected | - | 1.20 | <0.001 |
| Two weeks | **Two weeks** | 1450.9 | 0.872 | Not selected | - | 1.20 | <0.001 |
| Three weeks | **Two weeks** | 1450.9 | 0.872 | Not selected | - | 1.20 | <0.001 |
| Four weeks | **Two weeks** | 1450.9 | 0.872 | Not selected | - | 1.20 | <0.001 |
| Five weeks | **Two weeks** | 1450.9 | 0.872 | Not selected | - | 1.20 | <0.001 |
| Six weeks | **Two weeks** | 1450.9 | 0.872 | Not selected | - | 1.20 | <0.001 |

**Supplemental Table 4. *Model 1* sensitivity analysis with feature selection: Associations with cardiac arrest and acute coronary syndrome calls among those aged 16-39 using a negative binomial regression model with stepwise BIC feature selection, while varying the count period of the cumulative count of new COVID-19 cases covariate.**

| Count period for the cumulative count of new COVID-19 cases covariate | Count period for the cumulative count of 1^st^ and 2^nd^ vaccine doses covariate | Model BIC | Model Adjusted R^2^ | New COVID-19 case count covariate | | Cumulative count of vaccine doses covariate | |
| --- | --- | --- | --- | --- | --- | --- | --- |
|  |  |  |  | **Model beta coefficient** | **P-value** | **Model beta coefficient** | **P-value** |
| One week (current week) | **Two weeks** | 1463.3 | 0.874 | 8.60 | 0.339 | 0.866 | <0.05 |
| Two weeks | **Two weeks** | 1463.5 | 0.874 | 4.16 | 0.381 | 0.845 | <0.05 |
| Three weeks | **Two weeks** | 1463.3 | 0.874 | 3.29 | 0.330 | 0.789 | <0.05 |
| Four weeks | **Two weeks** | 1463.1 | 0.875 | 2.91 | 0.286 | 0.720 | 0.071 |
| Five weeks | **Two weeks** | 1463.0 | 0.875 | 2.61 | 0.263 | 0.654 | 0.127 |
| Six weeks | **Two weeks** | 1462.8 | 0.875 | 2.49 | 0.232 | 0.576 | 0.210 |

**Supplemental Table 5. *Model 1* sensitivity analysis without feature selection: Associations with cardiac arrest and acute coronary syndrome calls among those aged 16-39 using a negative binomial regression model without stepwise BIC feature selection, while varying the count period of the cumulative count of new COVID-19 cases covariate.**

| Count period for the cumulative count of new COVID-19 cases covariate | Count period for the cumulative count of 1^st^ and 2^nd^ vaccine doses covariate | Model BIC | Model Adjusted R^2^ | New COVID-19 case count covariate | | Cumulative count of vaccine doses covariate | |
| --- | --- | --- | --- | --- | --- | --- | --- |
|  |  |  |  | **Model beta coefficient** | **P-value** | **Model beta coefficient** | **P-value** |
| One week (current week) | **Two weeks** | 1844.8 | 0.937 | Not selected | - | 0.580 | P<0.001 |
| Two weeks | **Two weeks** | 1844.8 | 0.937 | Not selected | - | 0.580 | P<0.001 |
| Three weeks | **Two weeks** | 1844.8 | 0.937 | Not selected | - | 0.580 | P<0.001 |
| Four weeks | **Two weeks** | 1844.8 | 0.937 | Not selected | - | 0.580 | P<0.001 |
| Five weeks | **Two weeks** | 1844.8 | 0.937 | Not selected | - | 0.580 | P<0.001 |
| Six weeks | **Two weeks** | 1844.8 | 0.937 | Not selected | - | 0.580 | P<0.001 |

**Supplemental Table 6. *Model 2* sensitivity analysis with feature selection: Associations with cardiac arrest calls among all ages using a negative binomial regression model with stepwise BIC feature selection, while varying the count period of the cumulative count of new COVID-19 cases covariate.**

| Count period for the cumulative count of new COVID-19 cases covariate | Count period for the cumulative count of 1 and 2^nd^ vaccine doses covariate | Model BIC | Model Adjusted R^2^ | New COVID-19 case count covariate | | Cumulative count of vaccine doses covariate | |
| --- | --- | --- | --- | --- | --- | --- | --- |
|  |  |  |  | **Model coefficient** | **P-value** | **Model coefficient** | **P-value** |
| One week (current week) | **Two weeks** | 1859.7 | 0.938 | 0.23 | 0.984 | 0.699 | <0.001 |
| Two weeks | **Two weeks** | 1859.6 | 0.938 | 1.21 | 0.843 | 0.683 | <0.001 |
| Three weeks | **Two weeks** | 1859.5 | 0.938 | 1.70 | 0.685 | 0.662 | <0.001 |
| Four weeks | **Two weeks** | 1859.3 | 0.939 | 1.80 | 0.574 | 0.647 | <0.001 |
| Five weeks | **Two weeks** | 1859.2 | 0.939 | 1.75 | 0.492 | 0.638 | <0.001 |
| Six weeks | **Two weeks** | 1859.1 | 0.939 | 1.59 | 0.448 | 0.636 | <0.001 |

**Supplemental Table 7. *Model 2* sensitivity analysis without feature selection: Associations with cardiac arrest calls among all ages using a negative binomial regression model, without stepwise BIC feature selection, while varying the count period of the cumulative count of new COVID-19 cases covariate.**
